# The suppression of TdMRP3 genes reduces the phytic acid and increases the nutrient accumulation in durum wheat grain

**DOI:** 10.3389/fpls.2023.1079559

**Published:** 2023-01-18

**Authors:** Arianna Frittelli, Ermelinda Botticella, Samuela Palombieri, Stefania Masci, Silvia Celletti, Maria Chiara Fontanella, Stefania Astolfi, Pasquale De Vita, Mirko Volpato, Francesco Sestili

**Affiliations:** ^1^ Department of Agriculture and Forest Science (DAFNE), University of Tuscia, Viterbo, Italy; ^2^ Institute of Sciences of Food Production (ISPA), National Research Council (CNR), Lecce, Italy; ^3^ Department for Sustainable Process, Faculty of Agriculture, Food and Environmental Science (DiSTAS), Università Cattolica, Piacenza, Italy; ^4^ Council for Agricultural Research and Economics, Research Centre for Cereal and Industrial Crops (CREA-CI), Foggia, Italy; ^5^ Grandi Molini Italiani, Venezia, Italy

**Keywords:** durum wheat, genetic biofortification, micronutrients, mutagenesis, phytic acid, tilling

## Abstract

Micronutrient malnutrition affects more than half of the world population. Reduced bioavailability of microelements in the raw materials is considered one of the main causes of mineral deficiency in populations whose diet is largely based on the consumption of staple crops. In this context, the production of low phytic acid (*lpa*) cereals is a main goal of the breeding programs, as phytic acid (PA) binds essential mineral cations such as iron (Fe), zinc (Zn), manganese (Mn), potassium (K), calcium (Ca) and magnesium (Mg) precipitating in the form of phytate salts poorly digested by monogastric animals, including humans, due to the lack of phytases in the digestive tract. Since PA limits the bioavailability of microelements, it is widely recognized as an anti-nutritional compound. A Targeting Induced Local Lesions IN Genomes (TILLING) approach has been undertaken to silence the genes encoding the TdABCC13 proteins, known as Multidrug-Resistance associated Proteins 3 (TdMRP3), transporters involved in the accumulation of PA inside the vacuole in durum wheat. The TdMRP3 complete null genotypes showed a significant reduction in the content of PA and were able to accumulate a higher amount of essential micronutrients (Fe, Zn, Mn) compared to the control. The number of spikelets and seeds per spike, traits associated with the agronomic performances, were reduced compared to the control, but the negative effect was in part balanced by the increased grain weight. The TdMRP3 mutant lines showed morphological differences in the root apparatus such as a significant decrease in the number of root tips, root length, volume and surface area and an increase in root average diameter compared to the control plants. These materials represent a promising basis for obtaining new commercial durum wheats with higher nutritional value.

## 1 Introduction

Wheat, along with rice and maize, is one of three major cereals cultivated worldwide. The adaptability to a wide range of conditions, the good nutritional profile, along with its unique dough visco-elastic properties are the main reasons for its success (Shewry, 2009).

Durum wheat (*Triticum turgidum ssp. Durum*), the second wheat species most cultivated worldwide, is a key raw material in a wide variety of traditional foods largely consumed in the Mediterranean basin as a part of a diet style recognized as one of the healthiest in absolute terms (Willett et al., 1995; Sofi et al., 2010).

Where diets rely on plant-derived foods, it is crucial to increase the amount of health-promoting compounds (i.e. fibres, proteins, vitamins, antioxidants, minerals) in durum wheat as a valuable strategy to maintain good health and to prevent important non-communicable diet-related diseases (obesity, diabetes, cardiovascular disorders, osteoporosis, cancer), along with those associated to “hidden hunger”. In this regard, hidden hunger indicates a particular form of undernutrition that occurs when the intake and absorption of micronutrients are not enough for the daily requirement. This pathology affects almost one-third of the population, triggering serious diseases such as anaemia, weak bones, fatigue and weakened immune system and a few hidden impacts on the general well-being, lowering the life quality and increasing the risk of new pathologies (Lockyer et al., 2018; Vitamin and Mineral Nutrition Information System (VMNIS), 2022).

Genetic biofortification of staple crops represents an effective and sustainable strategy to boost essential minerals (i.e. iron, magnesium, calcium, potassium and zinc) in the human diet as it does not require the addition of fertilizers during the cultivation or additives in food production giving, as result, a stable over generations crop enriched in the target compound (Vasconcelos et al., 2017; Roberts and Mattoo, 2019).

*Myo-inositol-1,2,3,4,5,6-hexakisphosphate* (InsP_6_) is a ubiquitous component of eukaryotic cells that plays several regulatory roles (Shears, 2001). Also known as phytic acid (PA), it is the major phosphorus storage sink within the plant seeds and other plant tissues and organs such as pollen, roots, tubers and turions. The amount and distribution of PA within the kernel depend on the plant species. In barley, rice and wheat about 80% of PA is stored in the aleuronic layer and bran, whereas in maize and Arabidopsis mainly in the embryo and scutellum (O'Dell et al., 1972). Differently from cereals and Arabidopsis, about 95% of PA is accumulated in the cotyledons in legumes (Ariza-Nieto et al., 2007).

Due to its negative charges, PA binds important mineral cations such as zinc, iron, potassium, magnesium and calcium precipitating in the form of phytate salts poorly digested by monogastric animals, including humans, due to the lack of phytases inside the digestive tract. As it chelates ions the bioavailability of phosphorus and minerals is decreased and PA is considered an anti-nutritional compound. In addition, the excretion of undigested phosphate contributes to environmental pollution by accelerating the eutrophication of the soil (Raboy, 2009; Secco et al., 2017).

In plants, phytic acid biosynthesis is carried out through two metabolic pathways (Sparvoli and Cominelli, 2015). The lipid-dependent pathway acts in all plant tissues, while the lipid-independent pathway is mainly active in seeds. In the first step, glucose-6-phosphate is converted into myo-inositol-3-phosphate (Ins (3) P_1_) by myo-inositol-3-phosphate synthase (MIPS). The subsequent steps involve sequential phosphorylation of the inositol ring through various enzymes (inositol phosphate kinase 2, IPK2; inositol 1,3,4-trisphosphate 5-/6 kinase, ITPK; inositol polyphosphate 2- kinase, IPK1). The synthesized phytic acid is accumulated into globoids, spherical inclusions found within protein bodies (Krishnan, 2008; Regvar et al., 2011), and stored inside the vacuoles where it is transported by specific PA protein transporters, such as the *Multidrug-Resistance associated Proteins* (MRPs) (Sparvoli and Cominelli, 2014; Colombo et al., 2020; Cominelli et al., 2020a).

Previous breeding programs focused on the reduction of PA to increase the mineral content of food and the sustainability of agricultural production in different crops (Raboy, 2020). *Low phytic acid* (*lpa*) genotypes have been produced in all major grain crops using different strategies. In this regard, *lpa* mutants can be divided into three classes according to the step where the mutations affect the PA biosynthetic pathway or transport: 1) the first biosynthetic step in which glucose-6-phosphate is converted into Ins(3)P_1_ by MIPS; 2) the last biosynthetic step in which IPK1 phosphorylates InsP_5_ in the 2-position to synthesize PA; 3) the transport and storage of phytic acid into the vacuole through the targeting of MRP transporters (Sparvoli and Cominelli, 2015; Colombo et al., 2020).

In bread wheat TaMRP3 homeoalleles are located on the long arm of the 5A, 4B and 4D chromosomes and encode multidrug resistance-associated proteins belonging to the ABCC cluster of plant ATP-binding cassette (ABC) transporters (Bhati et al., 2014). MRP proteins are characterized by a common structure consisting of two soluble nucleotide-binding domains (NBD1 and NBD2), two hydrophobic transmembrane domains (TMD1 and TMD2) and an additional hydrophobic N-terminal extension (TMD0) connected by a cytosolic loop to the rest of protein (Sparvoli and Cominelli, 2014; Colombo et al., 2020). *Lpa* mutants were produced by targeting *MRP* genes in *Arabidopsis thaliana*, rice, soybean and common beans (Colombo et al., 2020), demonstrating that MRP proteins are involved in the transport of PA within the vacuole (Shi et al., 2007; Nagy et al., 2009).

Interestingly, Bhati et al., 2016 previously reported a partial suppression of *TaMRP3* genes by RNA interference (RNAi) and demonstrated its functional role in bread wheat. Due to its central role in PA transport, TdMRP3 is an attractive target for increasing the accumulation of minerals in durum wheat through a non-transgenic genetic approach focused on its inactivation.

In this paper, the genes encoding TdABCC13 (TdMRP3) were completely disrupted at DNA level through a Targeting Induced Local Lesions in Genomes (TILLING) strategy, a highly processive non-transgenic reverse genetics technique that combines chemical mutagenesis with a PCR-based screening for the identification of mutations in the gene of interest (McCallum et al., 2000).

We show that the effect of *TdMRP3* silencing was a significant reduction in PA content, resulting in an improved capability to accumulate micronutrients (Fe, Zn, Mn) in wheat seeds. In addition, it was tested whether the suppression of *TdMRP3* genes in durum wheat generates modifications in root system architecture with significant consequences on root ability to acquire water and nutrients.

To the best of our knowledge, this study represents the first example of a genetic approach that successfully reduced the accumulation of phytic acid and increased the bioavailability of essential minerals in durum wheat kernel.

## 2 Material and methods

### 2.1 Isolation of genes coding TdMRP3 from genomic databases

To identify the sequences of the *TdMRP3* genes, the orthologous genes of *T. aestivum* (TraesCS5A02G512500 and TreasCS4B02G343800) were used as queries in two independent approaches. In the first approach the two queries were blasted against *Triticum turgidum* ssp *durum* cv Svevo genome in Ensembl Plants database (https://plants.ensembl.org/index.html).

In the second one the orthologous query sequences were individually blasted against the Svevo Platinum genome available at the Svevo Platinum Genome Consortium (data unpublished). The exon/intron structure of the two identified durum wheat homeoalleles (*TdMRP3-A1* and *TdMRP3-B1*) was predicted by GENESCAN web tool (Burge and Karlin, 1997).

### 2.2 Bioinformatic analysis of TdMRP3 proteins

Domain topology and organization were predicted by analyzing and comparing the full-length amino acid sequence of each TdMRP3 protein in the UniProt database.

The orthologous sequences of the major grass species were identified by blasting the TdMRP3 protein sequences in NCBI BLASTP software (https://blast.ncbi.nlm.nih.gov/Blast.cgi). The orthologous sequence of *Arabidopsis thaliana*, used as an outsider, was isolated from TAIR database.

The multiple sequence alignment of MRP proteins (orthologous of TdMRP3) was performed by accurate Multiple Sequence Alignment (MSA) with PSI-Coffee 11.0 tool (Chang et al., 2012).

The phylogenetic analysis of MRP proteins was carried out by MEGA11 software (Tamura et al., 2021). The evolutionary history and distance among the selected taxa were deduced using the Neighbor-Joining (NJ) method with p-distance method and pairwise deletion option. The bootstrap consensus tree was inferred from 1000 replicates.

### 2.3 Plant materials

A preliminary *in silico* study allowed the identification of two durum wheat mutant lines possessing deleterious mutations on the two *TdMRP3* homeoalleles through the platform WheatTILLING available at the University of Davis (https://dubcovskylab.ucdavis.edu/home; Krasileva et al., 2017). In detail, the line Kronos 3179 has a splice site mutation located in the 5’ region of the intron 4 of *TdMRP3-A1*, while the line Kronos 4443 has a nonsense mutation in the exon 4 of *TdMRP3-B1* (**Supplementary Figure S1**). The presence of the two mutations was confirmed by Sanger sequencing.

The pyramiding of the two mutations was carried out by crossing the identified mutant lines TdMRP3-A1^-^ and TdMRP3-B1^-^. The partial and complete null mutants along with the controls (cv Kronos and wild-type sib lines derived by the cross) were grown in a controlled growth chamber with initial vernalization at 4-5°C for 3 weeks, followed by 18-26°C day and 16-18°C night temperature with a 16 h light period.

### 2.4 DNA extraction

Genomic DNA was extracted from leaves using the commercial kit NucleoSpin^®^ Plant II (Macherey-Nagel, Düren, Germany) according to the manufacturer’s instructions. The DNA was used as template for the genotyping analysis.

### 2.5 High resolution melting genotyping

High Resolution Melting (HRM) analysis was performed on *TdMRP3* gene amplicons including the targeted mutations, amplified from the genomic DNA of F_2_ progeny of the cross described in the “Plant materials” paragraph. Amplicons were produced by a nested PCR strategy. The first PCR was carried out to amplify genome-specific fragments of 514 bp and 409 bp for *TdMRP3-A1* and *TdMRP3-B1* homeoalleles, respectively, using the primer pairs reported in **Supplementary Table S1**. The reaction was performed in a 20 µl volume using the following conditions: 10 µl of 2X GoTaq^®^ G2 Hot Start Colorless Master Mix (Promega, Madison, USA), 0.5 µM of each primer, 20 ng of template DNA and nuclease-free water up to 20 µl volume, with the following conditions: 95°C for 2 min, followed by 38 cycles at 95°C for 30 s, 58°C for 30 s, 72°C for 1 min and a final extension at 72°C for 5 min. The first PCR reaction was diluted 60-fold and 2 µl were used as a template for the second PCR reaction for HRM analysis. The second reaction was carried out using the primer pairs reported in **Supplementary Table S1** and was prepared as follows: 2 µl of diluted DNA template, 5 µl of 2X GoTaq^®^ G2 Hot Start Colorless Master Mix (Promega), 0.5 µM of each primer, 1 µL LC Green Plus (Idaho Technology Inc., Salt Lake City, USA) and nuclease-free water up to 10 µl volume. The PCR program was carried out following these conditions: 95°C for 2 min, followed by 39 cycles at 95°C for 30 s, 60°C for 20 s, 72°C for 20 s and a final extension at 72°C for 5 min. At the end of the final extension step, the reaction was held at 95°C for 30 s, then at 25°C for 60 s. The PCR reaction was carried out in 96-well Frame-Star plates (4titude Ltd., Surrey, UK) overlaid with 10 µl of mineral oil (Sigma-Aldrich, St. Louis, MO, USA). The Light Scanner instrument (Idaho Technology, Inc.) was used to analyse the melting curves.

### 2.6 Determination of phytic acid

PA was determined in mature seeds of the selected F_4_ partial and complete null mutant lines (MRP3-A1^-^, MRP3-B1^-^ and MRP3-A1^–^B1^-^) along with the controls (cv. Kronos and WT sibling lines) using a commercial kit (K-PHYT kit, Megazyme Inc, Bray, Ireland). The grains were grounded to fine powder by a laboratory Cycline Mill (Cyclotec 1093, FOSS, Hilleröd, Sweden). One gram of the powder was suspended in 20 ml of 0.66 M HCl with continuous stirring overnight. The supernatant was then used for the colorimetric procedure according to the manufacturer’s protocol. PA content has been expressed in the form of mean values of three biological replicates ± standard error. Three technical replicates were carried out for each biological replicate. Significant differences between mean values were identified by applying a one-way analysis of variance and the *post hoc* Tukey’s HSD test, p-value< 0.01.

### 2.7 Visualization of iron deposits in seeds using the Perls stain

The *Perls* method is used to determine the localization of ferric iron (Fe^3+^) deposits stained in blue in the tissues (Krishnan et al., 2001; Prom-u-Thai et al., 2003). Briefly, the mature F_4_ seeds were soaked in dH_2_O for 5 hours. Then, the seeds were cut transversely and longitudinally and placed in Petri dishes, submerged in freshly prepared Perls staining solution (2% hydrochloric acid mixed with 2% potassium ferrocyanide) for 30 minutes. The seeds were then gently washed continuously in dH_2_O for 5 minutes. The intensity of staining was rated under a stereo microscope.

### 2.8 Determination of nutrients concentration

Mature F_4_ seeds of the selected mutant lines along with controls (cv. Kronos and WT sibling lines) were grounded to fine powder and oven-dried at 80°C to constant weight. Samples were introduced in polypropylene tubes (digiTUBES, SCP Science, Champlain, NY, USA) with 3 mL of concentrated nitric acid and 1 mL of concentrated hydrogen peroxide and heated in a block system (DIGIPREP, SCP Science, Champlain, NY, USA) for 120 min at 95°C. After digestion, the extracts were filtered by a 0.45 μm teflon filter (DigiFILTER, SCP Science, Champlain, NY, USA). After cooling down, the digests were diluted with dH_2_O and analyzed by inductively coupled plasma-mass spectrometry (ICP-MS 7900, Agilent Technologies, Santa Clara, CA, USA) with Octopole Reaction System (ORS). Phosphorous and sulfur in the digested solutions were determined by inductively coupled plasma-optical emission spectrometer (ICP-OES 5100 Agilent Technologies, Santa Clara, CA, USA). Data have been expressed as mean values of three biological replicates ± standard error. Three technical replicates were carried out for each biological replicate. The ICP-MS operating conditions are summarized in **Supplementary Table S2**.

### 2.9 Analysis of root morphological traits

The mature F_4_ seeds of the selected mutant lines along with control plants (cv. Kronos and WT sibling lines) were soaked in dH_2_O for 1 hour. Then they were transferred in Petri dishes and left to germinate for 5 days in the dark at room temperature. After germination, uniform seedlings were transferred to a plastic pot filled with 2 L of a continuously aerated nutrient solution (Celletti et al., 2016) and were placed in a growth chamber under 27/20°C and 14/10 h day/night cycles with a relative humidity of 80% and 200 μmol m^-2^ s^-1^ PAR at leaf level for 5 days. Roots were excised from the stem and subsequently placed in a Perspex tray with a shallow film of water to minimize root overlapping. Root systems were analysed using the WinRHIZO™ scanning equipment and software (EPSON1680, WinRHIZO Pro2003b Software; Regent Instruments Inc., Quebec, Canada) to determine their volume and surface area, total root length, root diameter and number of root tips. Data have been expressed as mean values of three biological replicates ± standard error. Three technical replicates were carried out for each biological replicate.

### 2.10 Field trial evaluation of yield-related traits

The experimental field trial was carried out at CREA-CI (Foggia, Italy) during the 2021-2022 growing season using standard agronomic practices. For this experiment, grains from two different genotypes were used: wild-type (cv. Kronos) and double null TdMRP3 mutant. Each genotype was seeded in single plots, consisting of 1-m rows, 30 cm apart, with 30 germinating seeds per plot, and following a randomized complete block design with three replications. Plots were hand-harvested at maturity and yield-related traits (i.e. Spike weight, g; Spike length, cm; Spikelets number per spike; Kernels per spike and Kernel weight per spike, g), measured from ten randomly selected spikes per row, were recorded.

## 3 Results

### 3.1 In silico analysis of TdMRP3 transporters

A blast search of *TaMRP3-A1* and *TaMRP3-B1* (TraesCS5A02G512500 and TraesCS4B02G343800) run against the durum wheat genome in Ensembl Plants revealed a high identity with TRITD5Av1G244640 and TRITD4Bv1G193220 both coding for proteins with ATP binding and ABC-type transporter activity. The comparison of the deduced protein sequences with TaMRP3 proteins showed that both TdMRP3 proteins lack the TDM0 domain and present a shorter IPR011527 ABCC1_TM domain (data not shown).

A second blast of the orthologous *TaMRP3* homeoalleles was performed against the Svevo Platinum Genome and allowed the identification of two sequence hits with high identity values, located on the chromosomes 5A and 4B, respectively. The *TdMRP3* sequences of Svevo include 11 exons and 10 introns (**Supplementary Figures S1A**, **S2**). The deduced amino acid sequences contain 1510 and 1505 aa for TdMRP3-A1 and TdMRP3-B1, respectively; both proteins showed five domains in the forward orientation typical of ABCC transporters: TMD0-TMD1-NBD1_TMD2-NBD2 (**Supplementary Figures S1B**, **S3**). The analysis performed by I-TASSER 3D model program confirmed the presence of five transmembrane α-helices in TMD0, six α-helices in each TMD1 and TMD2 and the presence of Walker A and B motifs in the two cytosolic nucleotide-binding domains NBD1 and NBD2 (**Supplementary Figure S1B**).

**Figure 1:**
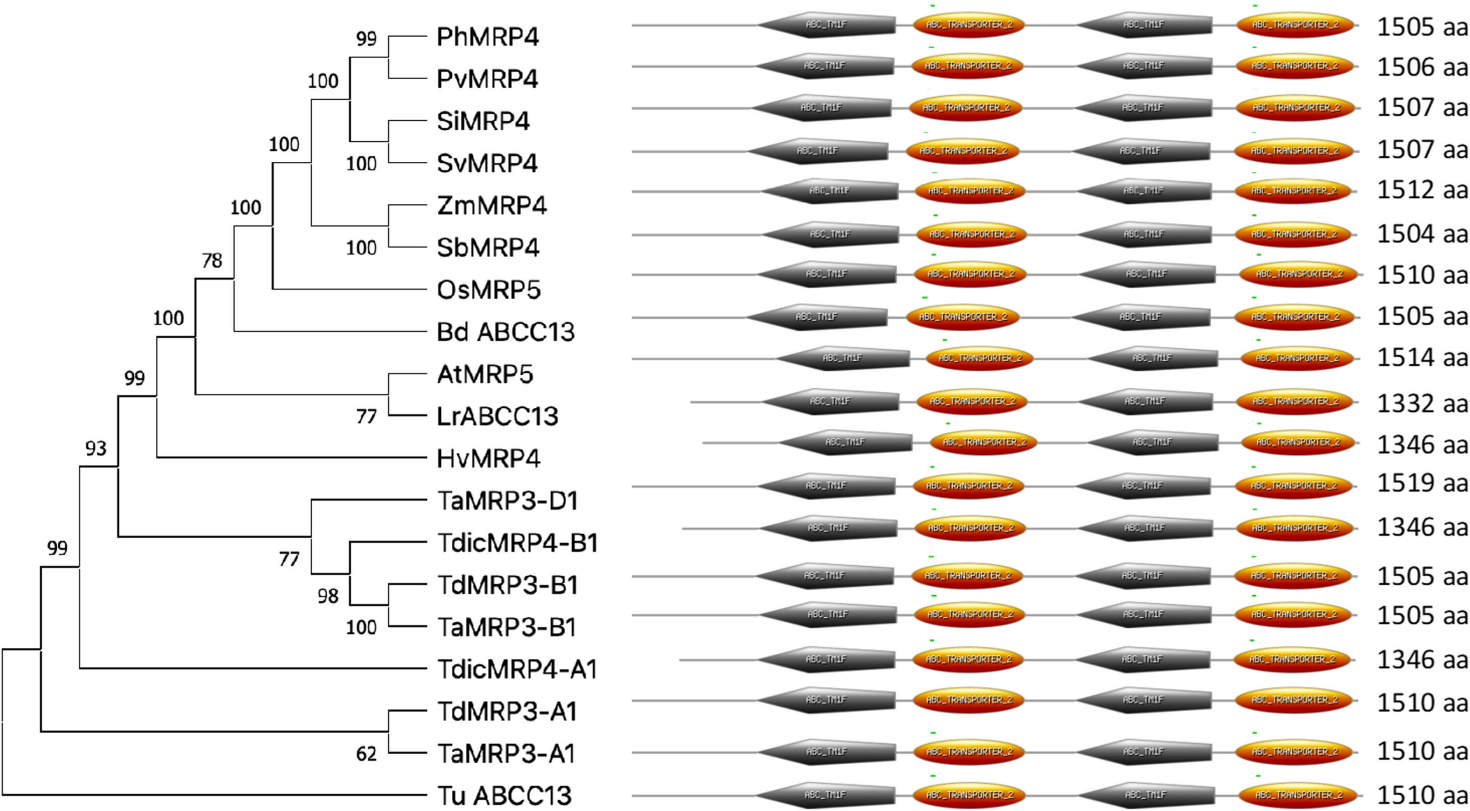
Phylogenetic tree of the ABCC multidrug resistance-associated protein (ABCC-MRP) in different plant species. Td: *Triticum turgidum ssp durum* MRP3-A1 (SVEVO PLATINUM SEQUENCE), MRP3-B (VEVO PLATINUM SEQUENCE), Ta: *Triticum aestivum* MRP3-A (XP_044384038 _TraesCS5A02G512500), MRP3-B (XP_044371728 TraesCS4B02G343800), MRP3-D (XP_044377018_TraesCS4D02G339000); Tu: *Triticum urartu* ABCC13 XP_048531007.1*;* Tdic: *Triticum dicoc*coides MRP4-A (XP_037437166.1), MRP4-B (XP_037426738.1); Hv: *Hordeum vulgare* MRP4 (XP 044983147.1); Bd: *Brachypodium distachyon* ABCC13 (XP_003558836.1); Zm: *Zea mays* MRP4 (EF586878); Sb: *Sorghum bicolor* MRP4 (XP 002468528.2); Pah: *Panicum hallii* MRP4 (XP 025794868.1); Pav: *Panicum virgatum* MRP4 (XP_039780041.1); Si: *Setaria italica* MRP4 (XP 004985744.1); Os: *Oryza sativa Japonica group* MRP5 (XP 015630971.1); At: *Arabidopsis thaliana* MRP5 (AT1G04120.1); Sv: *Setaria viridis* MRP4 (XP_034572896.1); Lr: *Lolium rigidum* ABCC13 like (XP_047089394.1). The tree constructed by the Neighbor-joining (NJ) method with pairwise deletion option and p-distance matrix in MEGA XI (Tamura et al., 2021). Bootstrap values (1000 replicates) were shown at each node.

A Multiple Sequence Alignment (MSA) of MRP proteins of major cereals and the model species *Arabidopsis thaliana* highlighted that the five domains of TdMRP3-A1 and TdMRP3-B1 were highly conserved among the orthologous proteins of the different species except for TDM0 (**Supplementary Figure S3**). In **Figure 1** the phylogenetic tree shows the evolutionary relationships of MRP transporters (orthologous of TdMRP3) among the grasses. TdMRP3 transporters showed phylogenetic proximity with other *Triticinae* (*Triticum aestivum*, *Triticum dicoccoides*) and barley (*Hordeum vulgare*). Noteworthy the amino acid sequences of the TdMRP3-A1 and TdMRP3-B1 were identical to those of bread wheat (TaMRP3-A1 and TaMRP3-B1, respectively). In addition, a higher homology was observed between the homeologous sequences of the genomes B and D compared to that of the A genome.

A second phylogenetic cluster was constituted by the MRP proteins isolated from *Oryza sativa*, *Zea mays*, *Sorghum bicolor*, *Panicum hallii*, *Panicum virgatum*, *Setalia italica*, *Setaria viritis*. Although the last cluster was more divergent from durum wheat sequences, the structure (domains) and the amino acid composition were strongly conserved among the different species considered for the phylogenetic tree (**Figure 1**; **Supplementary Figure S3**).

### 3.2 Identification and pyramiding of TdMRP3 mutations: selection of *lpa* mutants

Two knock out mutant lines for *TdMRP3* genes (one for each homeoallele), described in the “Material and methods” section, were identified through an *in silico* search on the “Wheat TILLING” platform (Krasileva et al., 2017). The single null mutant lines Kronos 3179 (TdMRP3-A1^-^) and Kronos 4443 (TdMRP3-B1^-^) were crossed to pyramid the two mutations and the F_2_ progenies genotyped by an HRM-genotyping assay (Wittwer et al., 2003), able to distinguish among heterozygous, homozygous and wild type genotypes for both the homeoalleles (**Supplementary Figure S4**). The analysis led to identifying four F_2_ homozygous double null mutants. In addition, three independent sister lines were identified for each single null mutant genotype (TdMRP3-A1^-^ and TdMRP3-B1^-^) and for the control line (wild type at each *MRP3* homeoallele-WT sibling lines). The different genotypes were confirmed by Sanger sequencing (**Supplementary Figure S4**).

### 3.3 Effect of the TdMRP3 suppression on the accumulation of phytic acid and nutrients in the kernel

**Figure 2:**
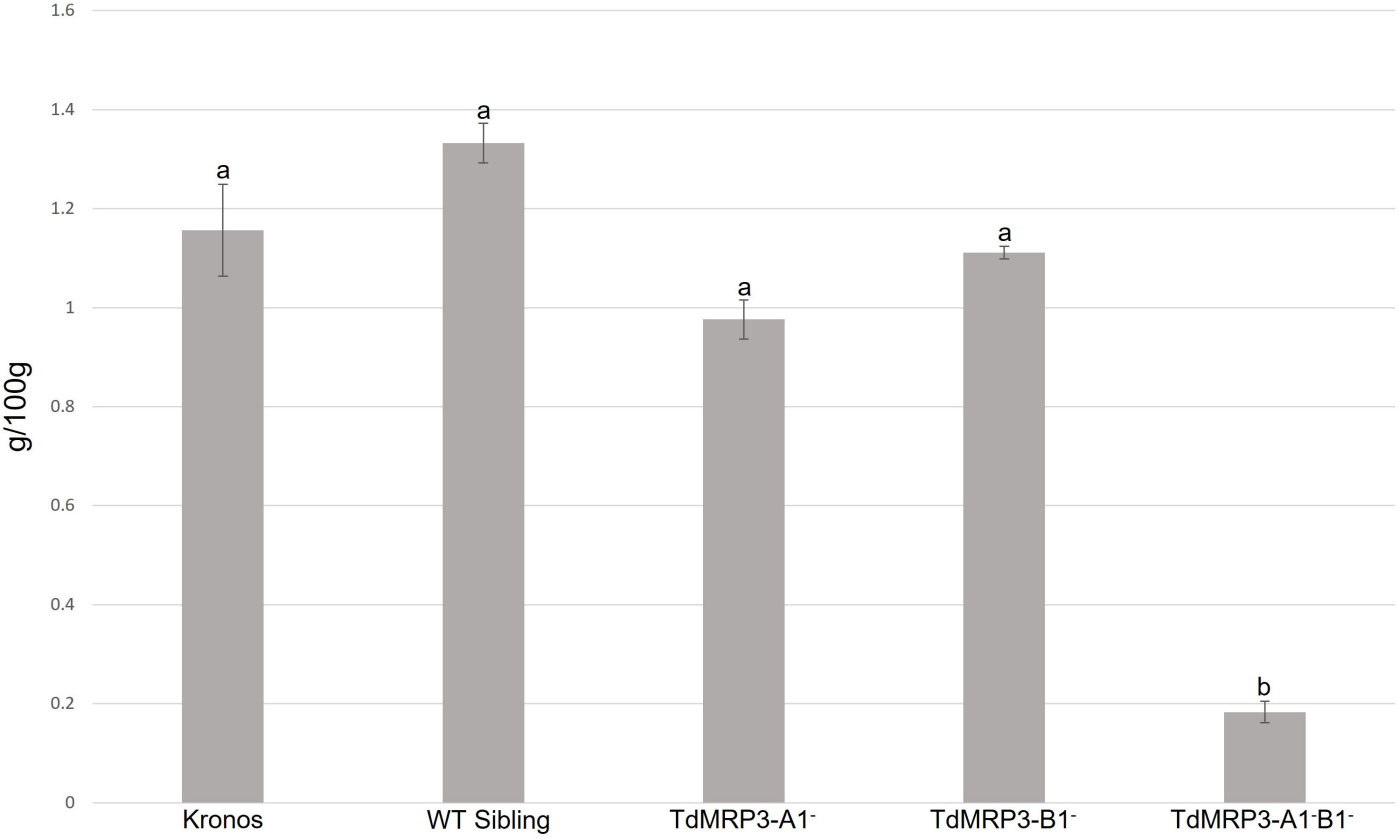
Phytic acid content in *lpa* mutants, WT sibling lines and cv. Kronos. Mean values of three biological replicates, error bars indicate standard error. Values followed by different letters differ signifcantly from one another (one-way ANOVA, Tukey HSD test, p<0.01).

To evaluate the effect of silencing of *TdMRP3* genes, mature grains were assessed for PA content in the whole set of mutants plus the controls. The complete null genotypes (TdMRP3-A1^-^B1^-^) showed a strong reduction in PA compared to the control (cv. Kronos) and WT sibling lines (-84.5% and – 86.5%, respectively), while no differences were observed in the single mutants (**Figure 2**).

**Figure 3:**
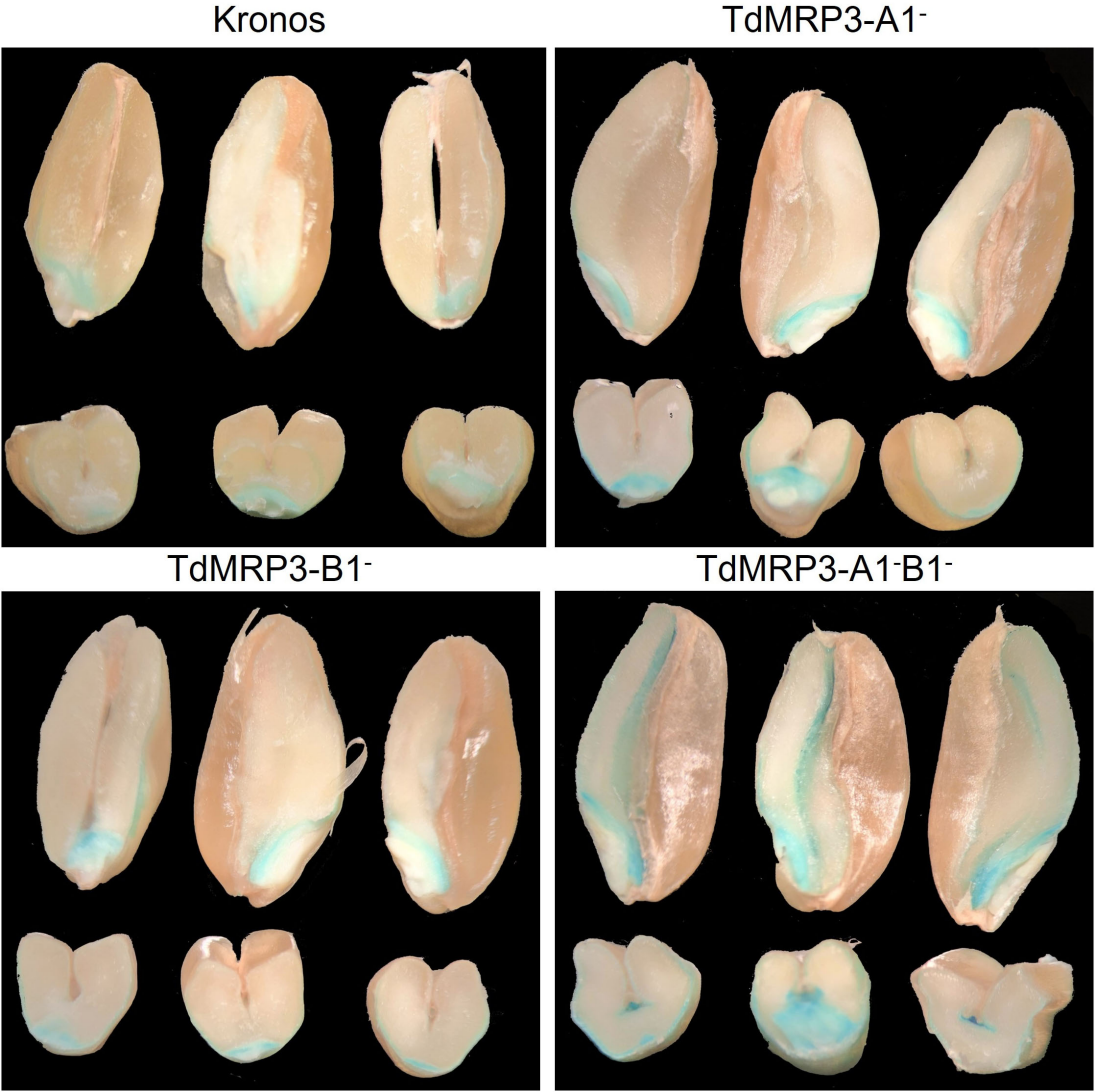
Visualization of iron deposits within the kernel using the *Perls* staining. In each image there are the longitudinal sections (top) and the transversal sections (bottom), of the seeds of cv. Kronos, TdMRP3-A1^-^, TdMRP3-B1^-^, TdMRP3-A1^-^B1^-^.

In light of the interaction between PA and cationic nutrients, the localization of iron deposits and micro/macronutrient accumulation were analysed in the mature seeds of the set of TdMRP3 mutants compared to the control. The intensity of iron deposits appeared strongly increased in the double null TdMRP3 mutants, interesting the scutellum, even more in the aleuronic layer and also evident in the endosperm (**Figure 3**). The single null genotypes (TdMRP3-A1^-^ and TdMRP3-B1^-^) also revealed visible differences in respect to the control, but less pronounced than the completely null genotypes (**Figure 3**).

**Figure 4:**
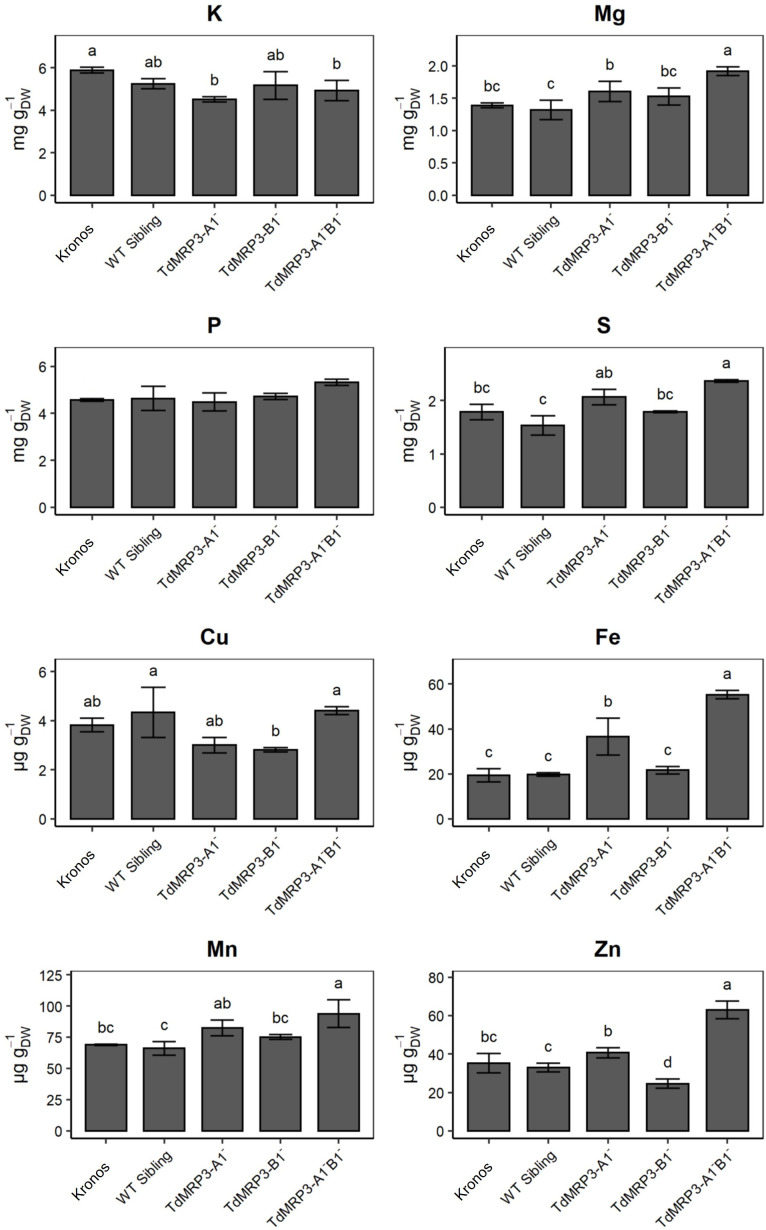
Concentration of nutrients in *lpa* mutants, WT sibling lines and cv. Kronos. Mean values of three biological replicates ± St. Dev. One-way ANOVA, LSD *post hoc* test, p <0.05.

The analysis of macro- and micro-nutrients in seeds highlighted differences among the genotypes. With regard to macronutrients, a higher accumulation of Mg and S was observed in the complete null TdMRP3 genotype compared to both the partial mutants and the controls (**Figure 4**). In detail, the whole grain of the complete null TdMRP3 genotypes showed a raise of 38.4% for Mg and 32.6% for S compared to the cv. Kronos.

No significant differences were detected for K in the mutant lines (either for partial or complete null genotypes), except for TdMRP3-A1^-^, which showed a slight decrease (-23.5%).

More interesting, a significant raise in micronutrient accumulation was recorded in the TdMRP3-A1^-^B1^-^ genotype (Fe +186.3%, Zn +78.4%, and Mn +36.3%), with the exception of Cu that maintained a concentration similar to the control (**Figure 4**). On the other hand, the accumulation pattern of micronutrients in partial genotypes was non-uniform. In particular, the accumulation of Fe did not change in the TdMRP3-B1^-^ mutant, whereas it increased by 89.3% in the other partial mutant TdMRP3-A1^-^. A different trend was also observed for Zn accumulation: it was reduced in the TdMRP3-B1^-^ genotype (-30.3%) and not significantly affected in TdMRP3-A1^-^.

### 3.4 Effects of TdMRP3 silencing on the agronomic performance and root morphology

**Figure 5:**
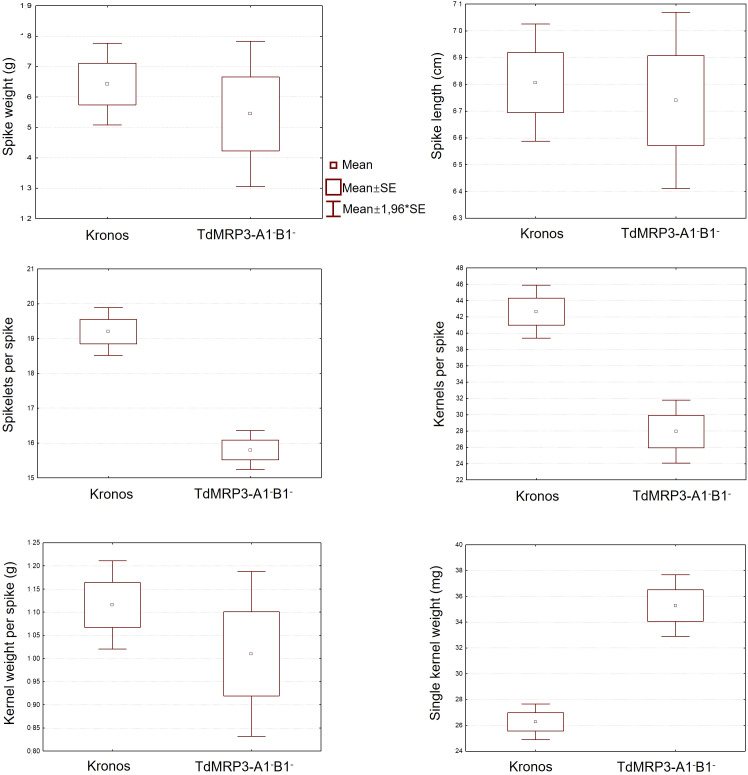
Agronomic traits analysis performed on: Spike weight (g), Spike length (cm), Spikelets per spike, Kernels per spike, Kernel weight per spike (g), Single kernel weight (mg). In each image there is the analysis of the seeds of cv. Kronos compared to TdMRP3-A1^-^B1^-^.

The agronomic performances of TdMRP3 mutant lines were evaluated in field by measuring a set of yield-related parameters of plant growth. No significant differences were registered for spike weight, spike length and kernel weight for spike between the control and TdMRP3 complete null mutant genotypes (**Figure 5**). Otherwise, TdMRP3-A1^-^B1^-^ mutant lines showed a reduction in the number of spikelets per spike (-17.7%) and a more significant reduction in the number of kernels per spike (-34.5%), while no significant differences for the grain weight per spike were recorded. Single kernel weight was increased by 34.2% in the TdMRP3-A1^-^B1^-^ mutant lines. No significant differences were observed between the single null lines and the wild type (data not shown).

**Figure 6:**
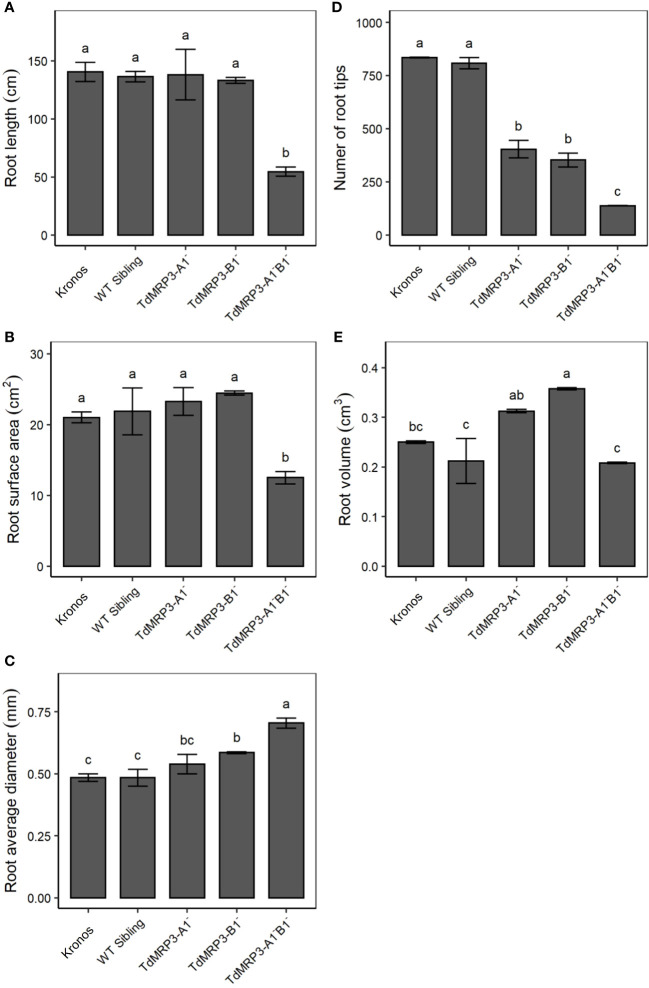
Root morphological traits: length **(A)**, surface area **(B)**, diameter **(C)**, number of root tips **(D)** and volume **(E)** in *lpa* mutants, WT sibling lines and cv. Kronos. Mean values of three biological replicates ± St. Dev. One-way ANOVA, LSD *post hoc* test, p <0.05.

As ABCC proteins are involved in several aspects of plant growth and root development, the root architecture of the set of mutant lines was analysed and compared to the control, considering the following parameters: root length, number of root tips, root surface area, volume and diameter (**Figure 6**). The mutant plants TdMRP3-A1^-^B1^-^ showed a significant decrease in the number of root tips (-83.5%), root length (-61.2%), volume (-16.8%), and surface area (-40.6%) compared to the cv. Kronos. A decrease in the number of root tips and an increase in root volume were observed in both the partial mutant genotypes, while no significant differences were detected in the root length and root surface area. Noteworthy, root average diameter significantly increased in all the mutants and increased by 45.8% in the TdMRP3-A1^-^B1^-^ (**Figure 6**).

## 4 Discussion and conclusions

Hidden hunger is a global health issue, that involves more than 3 billion people around the world, mostly in Africa, Asia, and Latin America. Diets poor in essential vitamins and minerals (micronutrients), typical of some countries of the above-cited continents, are the main driver of the hidden hunger. The development of biofortified staple crop varieties is an effective and low-cost strategy pursued by several major public and private sector organizations (such as Universities, INRA, CIMMYT and ICARDA) in Southern America, Asia and Africa. In this context, Harvest Plus is a big initiative focused on the development of bio-fortified wheat varieties, that involves partners from dozens of countries (Wani et al., 2022). Although numerous efforts have been focused on the realization of biofortified wheat genotypes, it remains still challenging.

In plants, PA is stored in the vacuole and functions as a Pi sink to aid plant growth upon seed germination. Due to its negative charge, PA chelates the cations forming poorly bioavailable phytate salts and limiting mineral ion bioavailability, thus promoting mineral deficiencies in the body (Grases et al., 2017; Samtiya et al., 2020). For this reason, boosting the bioavailability of minerals in plant food can be achieved by reducing PA. *Lpa* mutants can be produced by the impairment of the transport and storage of PA into the vacuole: in the cytosol PA is exposed to a dephosphorylation process carried out by cytosolic phosphatases, decreasing the final amount of phytates and increasing free Pi and cations (Sparvoli and Cominelli, 2015; Colombo et al., 2020). *Lpa* mutants were developed in wheat *via* ethyl methane sulfonate mutagenesis, and germplasm derived from these mutants was used for breeding purposes (Guttieri et al., 2004; Guttieri et al., 2006; Venegas et al., 2022).

Here, *lpa* mutants were generated in durum wheat targeting *TdMRP3* genes by TILLING, thereby causing the impairment of PA into the vacuole. TILLING strategy (McCallum et al., 2000) is a reverse genetics approach widely used in functional studies and breeding programs in numerous species, including bread and durum wheat (Slade et al., 2005; Botticella et al., 2011; Hazard et al., 2014; Sestili et al., 2015; Sestili et al., 2019; Garcia Molina et al., 2021) as it offers the big advantage to expand the genetic variability and to produce nontransgenic plants, that can be used for commercial purposes.

Our *in silico* analysis showed that the protein structure of MRP transporters was highly conserved among the major cereals. In detail, all the analysed sequences had the typical domains of ABCC transporters in forward orientation (Colombo et al., 2020), with a high identity degree in according to previous studies (Bhati et al., 2016; Cominelli et al., 2020b). Preliminary analysis on Ensembl database highlighted that durum wheat MRP3 proteins from Svevo genome v1 were different in length with respect to bread wheat and other cereal sequences. These durum wheat proteins missed an N-terminal region that included the TMD0 domain and part of the TMD1. Differently, the two durum wheat MRP3 sequences isolated by means of the new Svevo Platinum sequence showed the same length of TaMRP3 proteins (1510 and 1505 aa) and a perfect amino acid identity, sharing high phylogenetic proximity with other cereal species.

TdMRP3-A1 and -B1 were clustered into the same subgroup and were closer to the MRP proteins of *Triticinae* and barley and more divergent from other species, including the major cereals (rice and maize). Our data are in agreement with previous comparative studies that showed a high level of synteny in several gene loci among wheat, barley and other members of the *Triticeae* (Linde-Laursen et al., 1997; Mayer et al., 2011). Although the MRP3 sequences are highly conserved among the A, B, and D genomes of wheat, small amino acidic differences were identified. Unexpectedly a higher synteny was observed between the homeologous sequences encoded by the B and D genomes compared to MRP3-A1. In this regard, previous studies demonstrated higher synteny levels between A and D genome homeologues compared to those of the B genome (Akhunov et al., 2003; Pont et al., 2013).

In TdMRP3 double null mutants, PA was reduced by roughly 85% compared to the control in line with what was observed in *lpa* genotypes produced in legumes and other cereals, like maize and rice that showed a reduction of 80 and 90%, respectively (Silva et al., 2021). Differently, partial suppression of *TaMRP3* genes targeted by RNAi resulted in a modest reduction of PA content (in the range from -22 to -34%) in bread wheat. These differences can be explained by the different efficiency of silencing of the two approaches: full silencing in TILLING mutants, and reduction in the range of 40-72% of *TaMRP3* transcripts in the case of RNAi plants (Bhati et al., 2016). Accordingly, the accumulation of Fe, Zn and Mn in the grain was significantly raised in the TdMRP3 null mutants than in the transgenic RNAi lines (about +186% vs +18% for Fe; about +36% vs 0% for Mn; +78% vs +13% for Zn).

Generally, the phytic acid mutants described in many plant species were presented as agronomically poor for the low yields and for the poor germination capacity of the seeds (Raboy, 2020). In bread wheat, previous studies conducted on EMS mutants showed a reduction in PA of 30 to 40% (Guttieri et al., 2004), and a more contained loss of yield (from 8 to 25%).

In our study, the reduction in yield was comparable to bread wheat, although the reduction in PA was much higher (>85%). Actually, Guttieri et al. (2006) showed the absence of consistent effects on yield and yield-related traits in wheat *lpa* mutants, depending on the different genetic backgrounds in which the trait was transferred. In particular, in the soft white spring genetic background, the kernel weight of the *lpa* mutants was greater than that of the control wild type, confirming our findings, in which the partial reduction in yield, due to the reduction in the number of spikelets and kernels for spike, was compensated by a greater kernel weight. This suggested that the negative agronomic effects of the *lpa* genotype can be mitigated by adopting an adequate genetic improvement strategy.

In maize the suppression of *ZmMRP4* was associated with ungerminability and seed weight loss (Pilu et al., 2005; Cerino Badone et al., 2012). Pilu et al., 2005 found a decreased content of PA in maize mutant lines and showed that kernels with less than 20% of PA are unable to germinate. The same pleiotropic effects were reported in rice with the suppression of *OsMRP5*, orthologous of *TdMRP3* and *ZmMRP4* (Xu et al., 2009). In detail, the T-DNA knock line, in which *OsMRP5* was disrupted, showed a strong reduction of PA (>90%) and an inability to germinate.

Non-lethal *lpa* mutants were obtained in soybean and common bean through the silencing of the orthologous genes of *TdMRP3* (Oltmans et al., 2005; Cominelli et al., 2018). PA dropped between 75 and 90% in both the species but only in common bean no pleiotropic effect on yield traits and plant phenotype was observed.

The morphology of the root apparatus was markedly altered in our complete null TdMRP3 mutant line. In detail, the root length, volume and surface area and the number of tips, were decreased, while the root diameter was increased compared to the control plants. The morphological alteration of the root apparatus is a common adaptive strategy used by plants to cope with suboptimal nutrients (mainly nitrogen, sulfur and phosphate) and water availability (Aiken and Smucker, 1996; López-Bucio et al., 2003; Péret et al., 2011). The root diameter generally matches the elongation rate, which stops when the root reaches the minimal diameter (Pagès et al., 2020). Accordingly, shorter roots generally have a larger diameter.

It is well known that the plants face phosphate starvation by modifying the root system architecture through the inhibition of primary root growth, the increase of the lateral root formation and the growth and production of root hairs, thus allowing their root systems to efficiently utilize Pi from soils (Vance et al., 2003; Hinsinger et al., 2009; Péret et al., 2011). Besides soil P concentration, also plant Pi status can trigger morphological root responses (Vance et al., 2003; Lambers et al., 2006). Thus, the short-root phenotype showed by our mutants could be attributed to changes in Pi homeostasis induced by *TdMRP3* silencing resulting in increased availability of free phosphate in the cells and lower Pi demand from the soil. Moreover, MRP3 transporters are known to be expressed in several plant tissues including roots. So, we cannot rule out the possibility that the silencing of MRP3 transporter may have a more direct effect on anatomical root differences known to impact tolerance to Pi deficiency.

From a future perspective, it could be interesting to develop novel approaches able to limit the silencing of the *TdMRP3* expression to the seed to minimize the pleiotropic effects associated with *lpa* mutants. Bhati et al. (2016), using a different silencing technology, did not find a significant difference in the root length between the control and the RNAi transgenic plants. Moreover, they observed that the number of lateral roots was higher in the transgenic lines compared to the control plants. We could interpret these contrasting results by considering that the critical P supply and plant status for the activation of P-deficiency response, including root alterations, differ among plant species (Teng et al., 2013).

In conclusion, this study shed light on the functional role of TdMRP3 in durum wheat. In detail, it confirmed that the reduction of PA through the silencing of MRP transporters is a good strategy to obtain durum wheat genotypes biofortified in essential minerals and with acceptable agronomic performances. To date, to the best of our knowledge, this represents the first study in wheat allowing at the same time to increase the accumulation of essential minerals such as Zn, Fe and Mn.

Noteworthy, the plant lines can be used as starting material in breeding programs focused on mineral biofortification without any legal restriction associated with genetically modified organisms.

## Data availability statement

The datasets presented in this study can be found in online repositories. The names of the repository/repositories and accession number(s) can be found in the article/**Supplementary Material**.

## Author contributions

AF, SP, SC, MF and PV: investigation. EB and FS: conceptualization. FS and MV: funding acquisition. AF, SP and FS: data curation. SA, FS, PV and SM: visualization and validation. AF and FS: writing-original draft preparation. PV, EB, SA, SM and SM: writing-review & editing. FS: project administration. All authors contributed to the article and approved the submitted version.
