# Supplementary material for: The suppression of TdMRP3 genes reduces the phytic acid and increases the nutrient accumulation in durum wheat grain
Source: Front Plant Sci. 2023 Jan 18;14:1079559. doi: 10.3389/fpls.2023.1079559 (PMC9890658; doi:10.3389/fpls.2023.1079559)
Supplement: Supplementary file 1 [file DataSheet_1.pdf]

Supplementary Material

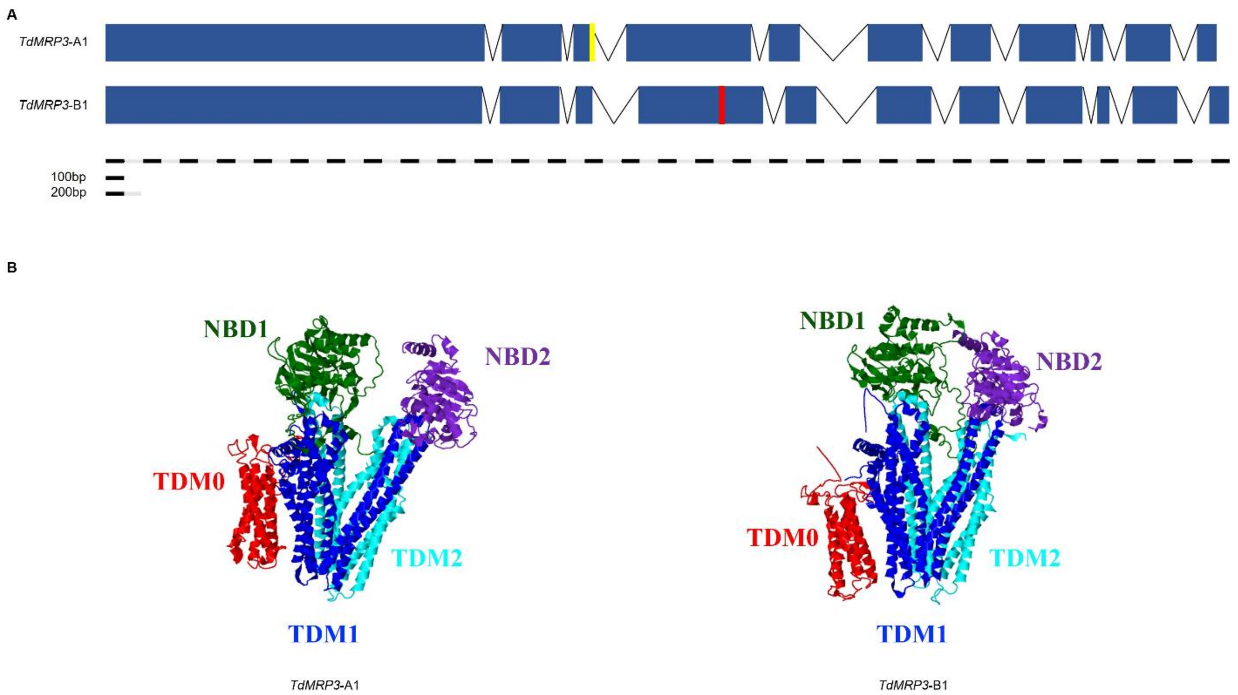

Supplementary Figure S1. *TdMRP3* gene and protein structures. (A) Gene structures of *TdMRP3* homeoalleles. Blue rectangles represent exons, the yellow box indicates the splice site mutation located in the 5′ region of the intron 4 on the homeoallele *TdMRP3-A1* (mutant line Kronos 3179). The red box indicates the premature stop codon located on the exon 4 of the homeoallele *TdMRP3-B1* (mutant line Kronos 4443). (B) Domain representation of modelled TdMRP3 protein [Colour code: Red-TMD0, Blue-TMD1, Green-NBD1, Light blue-TMD2, Violet-NBD2].

> TdMRP3-A1 gene

ATGCCGCGCCTCCGCGCCCACACCCCCCTCCCCCTCACGGAGGCCGCCGCCGCCGCCGCGCACGCCGCGCTGCTCGCCCTCGCGCTCCT
CCTCCTGCTCCTCCGCGGCGCGCGCGCCCTCGCCTCCCGCTGCGCCTCCTGCCTCAAGCCGCCCCGCCGCGCCCGCAACCTCGTCCACG
ATGGGCCGCCCCTCGCCTCCCCGCCGCCCGCCGCCGGGGGCGCCTGGTTCAGGGCCGCCCTCGCCTGCTGCGCCTACGTCCTGCTGGCG
CAGCTCGCCGCGCTGACCTACGAGGTCGCGGCCGCGCCGCCGCCCGTCGAGGCCGAGGCGCTGCTGCTGCCGGCCGTGCAGGCGCTGGC
CTGGGCGGCCCTGCTGGCGCTCGCGCTGCGGGCGCGCGCCGGGGGCCGCGGCAGGTTCCCGGCGCTCGTCCGGGTCTGGTGGGTGCTCG
CCTTCGCGCTCTCCGTCGCCATCGCCTTCGACGACTCCAGGCGCCTCATGGGCGCCGACGACCGTGATGCGGACTACGCGCACATGGTC
GCCAACTTCGCGTCGCTGCCGGCCCTCGGCTTCCTCTGCTTGGTTGGTGTGATGGGTTCCAGCGGTGTCGAGTTGGAGTTCAGTGATGA
CGACACCGGTGTCCACGAGCCCCTGTTGCTCGGCGGCCAGCGCAGAGGTGCCGAGGAGGAGCCCGGCTGCCTGCGGGTGACTCCCTACG
GCGATGCCGGGATCCTCAGCCTTGCAACGCTCTCATGGCTCAGTCCGCTGCTCTCGGTTGGGGCCAAGAGGCCGCTCGAGCTGGCTGAC
ATACCCTTGCTGGCTCACAAGGATCGTGCCAAGTTCTGCTACAAGGCCATGAGCAGTCACTATGAGCGCCAACGGCTGGAGTGCCCTGA
CAAGGAGCCATCGCTGGCATGGGCAATACTCAAGTCCTTCTGGCGGGAGGCGGCCATCAACGGCGCCTTCGCCGCGGTGAACACCGTCG
TGTCCTATGTCGGCCCCTACCTGATCAGCTACTTTGTGGACTACCTCAGTGGCAAAATTGCCTTCCCCCATGAAGGTTACATCCTTGCC
TCCGTATTTTTTGTATCAAAGTTGATTGAGACGCTCACTGCTCGCCAGTGGTACCTGGGCGTGGACGTCATGGGGATCCATGTCAAGTC
CGGGCTGACGGCCATGGTGTACCGGAAGGGCCTCAGGCTGTCGAATGCCTCAAAGCAGAGCCATACGAGCGGTGAGATTGTGAATTACA
TGGCGGTCGATGTGCAGCGGGTGGGGGACTATGCGTGGTACTTTCATGACATATGGATGCTTCCACTGCAGATCATCCTTGCGCTCGCC
ATCCTGTACAAGAATGTCGGGATCGCCACTGTCTCGACATTGATAGCCACCGCGCTGTCAATTGCTGCCTCGGTTCCTGTGGCGAAGCT
GCAGGAGCACTACCAAGATAAGCTGATGGCAGCAAAGGATGAGCGAATGCGCAAGACTGCAGAGTGCTTGAAGAGTATGAGAATTCTCA
AGTTGCAGGCATGGGAGGACCGGTATAGGATAATGCTTGAAGAGATGAGGAACGTTGAATGCAGGTGGCTCAAGTGGGCTTTGTACTCA
CAGGCCGCAGTTACGTTTGTTTTCTGGAGTTCGCCAATCTTTGTCTCGGTCATAACTTTCGGCACTTGTATATTGCTTGGTGGCGAGCT
CACTGCCGGAGGTGTTCTCTCTGCATTAGCTACCTTTAGGATCCTTCAAGAGCCTCTGAGGAATTTCCCTGATCTCATCTCCATGATAG
CTCAGACGAGGGTGTCTTTGGACAGGTTGTCTCACTTCTTACGGCAAGAAGAGTTGCCGGATGATGCAACAATAAGTGTTCCACAGGGT
AGTACAGACAAGGCAATCGATATCAGGGATGGCAGTTTCTCTTGGAACCCATCTTGCTCAAACCCTACGCTCTCTGATATACAACTTAG
TGTGGTGAGAGGCATGAGAGTAGCAGTCTGTGGTGTGATTGGTTCTGGCAAATCAAGTCTATTGTCCTCTATACTTGGGGAGATACCCA
AGCTGTCTGGCCAAGTAAGTACACGCATAAAAATAAAGAGAGTTGATGCTAACTTGAATGACAGTTTAATTACATTTTCCTTCTGCTCC
TTATTCAGGTTAGGATCAGTGGTACAGCAGCATATGTTTCTCAGACTGCCTGGATACAGTCTGGAAATATTGAGGAGAACGTTCTTTTC
GGCACTCCAATGGACAGACCGCGTTATAAGAGAGTACTTGAGGCTTGCTCCCTGAAGAAAGATCTTCAGTTGCTCCAGTATGGAGATCA
GACCATCATTGGTGACAGAGGCATTAATTTGAGTGGAGGTCAGAAACAGAGAGTGCAGCTTGCGAGAGCATTGTACCAAGATGCTGATA
TTTATTTGCTTGATGACCCCTTCAGTGCTGTTGATGCTCATACTGGAAGCGATCTATTTAAGGTCTGTATCGATGTTGCCTTATTCTAT
TTTTTTTAAAGGTGCTTCTGTTTCATAACATGTCAGATTTTGTGTTGCAGGACTATATATTGGGGGCACTAGCTAGCAAAACAGTAATT
TATGTAACCCATCAAGTCGAGTTCCTACCAGCTGCTGACTTGATATTGGTAATACTACTTTTCTCACAAAATCTATTAGCCTATTGAGT
TGTTCAATTTGACGTGTTGTTTATCCATGTTTCCTGATTAGAAAAACCAGCAGATTCTGCATGAAAGTTGCGCTATCTTTTCTAATACT
TCTCATTGGACTGTTCATGGGATAATGGGCCCTTGATCTCTCATTGTGTGTTTTTTTTTGTCATGGCACAGGTTCTTAAGGATGGTCAT
ATCACCCAAGCTGGAAAATATGATGATCTTCTCCAAGCTGGAACCGATTTCAATGCTCTGGTTTCTGCTCATAATGAAGCTATCGAGAC

CATGGATTTTGGCGAAGATTCTGATGGAGATATCGCTCCTTCCGTTCCTAACAAAAGATTGACACCAAGTGTTAGCAATATCGATAACC
TGAAAAATAAAGTATCTGAAAATGGGAAGTCATCTAATACACGTGGAATTAAGGACAAGAAAAAGAGTGAAGAACGTAAGAAGAAGCGT
ACTGTTCAGGAAGAGGAGAGGGAGCGAGGAAGAGTTAGCTTAAATGTTTATTTAACATACATGGGGGAAGCCTACAAAGGTTCACTGAT
TCCACTCATTGTCTTGGCGCAAACCCTGTTCCAAGTTCTTCAGATTGCCAGTAACTGGTGGATGGCATGGGCAAACCCACAAACAGAAG
GAGATGCACCTAAGACAAGTAGTGTGGTCCTTCTTGTTGTTTATATGTGCCTTGCTTTCGGGAGTTCATTGTTTGTGTTTGTGAGAAGC
CTTCTTGTAGCTACATTTGGTTTAGCAGCTGCTCAGAAACTATTTATAAAAATGCTAAGGTGTGTGTTTCGAGCGCCAATGTCATTCTT
CGATACTACACCATCTGGACGGATTCTGAATCGAGTAAGTTTAGATCATGCTCTTGCTCCTAATTTGTATTTGACACCTGTGTGGCTTT
TCTAATTTGCATACTCTAATCTTCTTCAATCATACTTCATATCAGGTTTCTGTAGATCAAAGTGTTGTGGACCTTGATATAGCATTCAG
GCTAGGGGGGTTTGCATCAACAACAATTCAACTCCTTGGAATCGTTGCTGTCATGAGCAAAGTCACATGGCAAGTTCTGTTTCTTATAG
TTCCTATGGCTATGGCATGCATGTGGATGCAGGTAAATTGCTAAACTTTCAGGTTTTCTTTTAGGGCTAACATTACCTGGGATCCTTAA
TTTCTTTAATGGAAATTGTAATACAAATAAGGCAGATAGCAAGGGTAGATACACCTAAATGGCTAAATGAATCACTAAAGAATTATGCG
CACATAATTTTTTTGGCTGCCTTTGTCATTTAACTAGCAGCCAATTAACCAGAGAACATATGCATATGTTCAGCATGAAAGTATCGAAA
GTTGTGAAGTAAGCTTATTATTCTTAAAGCACGAGGCCTGTGTTCAAGATAGCTTGATGATTTTGTGTACCAAAAGACTTTTAAAGATC
TTCATCACACAATAGATAATGGATGTCCTTGCTACCATGAACTTGCAGAGATATTACATTGCTTCATCGAGGGAGCTGACTAGGATCTT
GAGTGTTCAGAAGTCTCCAGTGATCCATTTGTTTAGCGAGTCAATCGCTGGTGCTGCTACTATCAGAGGATTTGGTCAGGAGAAAAGAT
TCATGAAAAGAAATCTTTACCTCCTTGATTGTTTTGCTCGGCCTTTGTTTTCCAGCCTTGCAGCTATTGAATGGCTCTGCCTGCGAATG
GAATTGCTCTCAACCTTTGTGTTTGCTTTCTGCATGGCAATACTTGTGAGCTTTCCTCCTGGAACAATTGAACCAAGTATGTCTTATCT
GCTGTCTCACACACCGTTCTGTTGCATTTTATGATCTTGCCTTACCCAATAGATCACTTGTTAGGTGCACTAGAAAACTTGCATATCTT
TTTCAATAAAATATGGCAATATGCTTCTCAATCTAAACAAGCTTTCCAATTGATAGGCATGGCTGGGCTTGCTGTCACATATGGACTCA
ACTTAAATGCTCGGATGTCAAGGTGGATATTGAGCTTCTGTAAATTAGAGAACAGAATCATCTCTGTTGAGCGTATTTATCAGTATTGC
AAGATTCCTAGTGAAGCACCACTGATTATCGAGAATTGCCGTCCCCCTTCCTCATGGCCTGAGAATGGAAACATTGAATTGATTGATCT
CAAGGTAAACTTAGCCCGTAGCCATTATTTATTTGTCATTATCCATTTGCTCTTCGTTATATATATTCACCAAAAGTAGTATGTAATCT
ATTTTTCTTCTTCTAAGCTGAACATACTTCTGTTTTATTTACGGCATCTTATTGTGCTTTTGCTAAATATCATAGGTTCGCTACAAGGA
TGACCTTCCCTTTGTTCTACATGGAGTCAGTTGTATTTTTCCTGGTGGGAAAAAGATTGGGATTGTAGGACGAACTGGAAGTGGTAAAT
CTACTCTCATTCAGGCTCTTTTCCGTCTAATTGAACCAGCAGGAGGGAAAATTATCATTGACAACATCGATGCTTCTGCGATTGGCCTT
CATGATCTGCGGTCACGGTTGAGCATCATTCCTCAGGACCCTACATTGTTCGAGGGTACTATCAGAATGAATCTTGACCCTCTTGAAGA
GCGTTCTGATCAAGAAATTTGGGAGGTATGCCATGGTCGCTTTTATGCTATCCTCATGTTAAATGTGCCATGATCTTTATGGAAGAGAA
TTCTTTTCTTTCAGGCACTAGAGAAGTGCCAGCTAGGAGAGGTCATTCGTTCGAAGGAAGAAAAACTGGACAGTCCAGGTTTGTTCTAA
TTTTGAATATTCAAACACTTCTTCAAAGAGTGAGCATGACACTTCGTAAAGCATGACACCTCGTAAAACAAAATTATATTTTCCTGGAT
ATTTGTGGTGCCATTGCAGTACTGGAGAATGGGGATAACTGGAGTGTGGGACAGCGCCAGCTTATTGCATTGGGTAGGGCGCTGCTTAA
GCAGGCAAGAATTTTGGTGCTTGATGAGGCAACGGCATCAGTTGACACAGCTACAGATAATCTCATCCAGAAGATCATCCGCAGCGAAT
TCAGGGATTGCACTGTCTGTACAATTGCACACAGGATCCCAACGGTTATCGACAGTGACCTAGTCATGGTGCTTAGCGACGGTACATGC
TCCTTCACACGATTAACCAAGACCATCACATGTTTTGTTTCTTACTGATCGTATGTTGAATTATCCGAGTAACACTCCTAGCTAAACAC

TGTTCTTGGAGCAACAGCCTTTACGGTGAATTCACCTTGTTCTTGTTTATTTCAGGTAAAATTGCGGAGTTCGACACACCCCAGAGGCT
TCTGGAGGACAAGTCCTCGATGTTCATGCAGCTAGTATCCGAGTACTCCACCAGGGCGAGCTGTATATAG

> TdMRP3-A1 protein

MPRLRAHTPLPLTEAAAAAAHAALLALALLLLLLRGARALASRCASCLKPPRRARNLVHDGPPLASPPPAAGGAWFRAALACCAYVLLA
QLAALTYEVAAAPPPVEAEALLLPAVQALAWAALLALALRARAGGRGRFPALVRVWWVLAFALSVAIAFDDSRRLMGADDRDADYAHMV
ANFASLPALGFLCLVGVMGSSGVELEFSDDDTGVHEPLLLGGQRRGAEEEPGCLRVTPYGDAGILSLATLSWLSPLLSVGAKRPLELAD
IPLLAHKDRAKFCYKAMSSHYERQRLECPDKEPSLAWAILKSFWREAAINGAFAAVNTVVSYVGPYLISYFVDYLSGKIAFPHEGYILA
SVFFVSKLIETLTARQWYLGVDVMGIHVKSGLTAMVYRKGLRLSNASKQSHTSGEIVNYMAVDVQRVGDYAWYFHDIWMLPLQIILALA
ILYKNVGIATVSTLIATALSIAASVPVAKLQEHYQDKLMAAKDERMRKTAECLKSMRILKLQAWEDRYRIMLEEMRNVECRWLKWALYS
QAAVTFVFWSSPIFVSVITFGTCILLGGELTAGGVLSALATFRILQEPLRNFPDLISMIAQTRVSLDRLSHFLRQEELPDDATISVPQG
STDKAIDIRDGSFSWNPSCSNPTLSDIQLSVVRGMRVAVCGVIGSGKSSLLSSILGEIPKLSGQVRISGTAAYVSQTAWIQSGNIEENV
LFGTPMDRPRYKRVLEACSLKKDLQLLQYGDQTIIGDRGINLSGGQKQRVQLARALYQDADIYLLDDPFSAVDAHTGSDLFKDYILGAL
ASKTVIYVTHQVEFLPAADLILVLKDGHITQAGKYDDLLQAGTDFNALVSAHNEAIETMDFGEDSDGDIAPSVPNKRLTPSVSNIDNLK
NKVSENGKSSNTRGIKDKKKSEERKKKRTVQEEERERGRVSLNVYLTYMGEAYKGSLIPLIVLAQTLFQVLQIASNWWMAWANPQTEGD
APKTSSVVLLVVYMCLAFGSSLFVFVRSLLVATFGLAAAQKLFIKMLRCVFRAPMSFFDTTPSGRILNRVSVDQSVVDLDIAFRLGGFA
STTIQLLGIVAVMSKVTWQVLFLIVPMAMACMWMQRYYIASSRELTRILSVQKSPVIHLFSESIAGAATIRGFGQEKRFMKRNLYLLDC
FARPLFSSLAAIEWLCLRMELLSTFVFAFCMAILVSFPPGTIEPSMAGLAVTYGLNLNARMSRWILSFCKLENRIISVERIYQYCKIPS
EAPLIIENCRPPSSWPENGNIELIDLKVRYKDDLPFVLHGVSCIFPGGKKIGIVGRTGSGKSTLIQALFRLIEPAGGKIIIDNIDASAI
GLHDLRSRLSIIPQDPTLFEGTIRMNLDPLEERSDQEIWEALEKCQLGEVIRSKEEKLDSPVLENGDNWSVGQRQLIALGRALLKQARI
LVLDEATASVDTATDNLIQKIIRSEFRDCTVCTIAHRIPTVIDSDLVMVLSDGKIAEFDTPQRLLEDKSSMFMQLVSEYSTRASC

> TdMRP3-B1 gene

ATGCCGCGCCGCGCCCTCCCCCTCGCCGAGGCCGCCGCCGCCGCCGCGCACGCCGCGCTGCTCGCCCTCGCGCTCCTCCTCCTGCTCCT
CCGCGGCGCGCGCGCCCTCGCCTCCCGCTGCGCCTCCTGCCTCAAGCCGCCCCGCCGCGCCCGCAACCCCGCCCTCGCCGGCGATGGGG
CGCCCCTCGCCCCCTCGCCGCCCGCCGCCGGGGGCGCCTGGTACCGGGCCGCCCTCGCCTGCTGCGCCTACGCCCTGCTGGCGCAGCTC
GCCGCGCTGAGCTACGAGGTCGCGGCCGCCGCGCCGCCCGCCGAGGCCGAGGCGCTGCTGCTGCCGGCCGTGCAGGCGCTGGCCTGGGC
GGCCCTGCTAGCGCTCGCGCTGCGGGCCCGCGCCGGCAGGTTCCCGGCGCTCGTGCGGGTCTGGTGGGTGCTCGCCTTCGCGCTCTCCC
TCGCCATCGCCTTCGACGACTCCAGGCGCCTCATGGGCGCCGACGACCACGATGCGGACTACGCGCACATGGTCGCCAACTTCGCGTCG
CTGCCGGCCCTCGGCTTCCTCTGCTTGGTTGGTGTGATGGGTTCCAGCGGTGTCGACTTGGAGTTTAGTGACGACGACACCGGTGTCCA
CGAGCCCCTGTTGCTCGGCGGGCAGCGCAGAGGCGCCGAGGAGGAGCCCGGCTGCCTGCGGGTGACTCCCTACGGCGACGCCGGGATCC
TCAGCCTTGCAACACTCTCATGGCTCAGTCCTTTGCTCTCGGTTGGGGCCAAGAGGCCGCTCGAGCTGGCTGACATACCCTTGCTGGCT
CACAAGGATCGTGCCAAGTTCTGCTACAAGGCCATGAGCAGTCACTATGAGCGCCAACGGCTGGAGTGCCCTGACAAGGAGCCGTCGCT
GGCATGGGCAATACTCAAGTCCTTCTGGCGGGAGGCGGCCATCAACGGCGCCTTCGCCGCGGTGAACACCGTCGTGTCCTATGTCGGCC
CCTACCTGATCAGCTACTTTGTGGACTACCTCAGTGGCAAAATTGCCTTCCCCCATGAAGGTTACATCCTTGCCTCAGTATTTTTCGTA
TCAAAGTTGATTGAGACGCTCACTGCTCGCCAGTGGTACCTGGGCGTGGACGTCATGGGGATCCATGTCAAGTCCGGGCTGACGGCCAT
GGTGTACCGGAAGGGCCTCAGGCTGTCGAATGCCTCAAAGCAGAGCCATACGAGCGGTGAGATTGTGAATTACATGGCGGTTGATGTGC
AGCGGGTGGGGGACTATGCATGGTACTTTCATGACATATGGATGCTTCCACTGCAGATCATCCTTGCGCTCGCCATCCTGTACAAGAAT
GTCGGGATCGCCACCGTCTCGACATTGATAGCCACCGCGCTGTCAATTGCTGCCTCGGTTCCTGTGGCCAAGCTGCAGGAACACTACCA
AGATAAGCTAATGGCAGCAAAGGATGAGCGAATGCGCAAGACTGCAGAGTGCTTGAAGAGTATGAGAATTCTCAAGCTGCAGGCATGGG
AGGACCGGTACAGGATAATGCTTGAAGAGATGAGGAACGTTGAATGCAGGTGGCTCAAGTGGGCTTTGTACTCGCAGGCCGCAGTTACG
TTTGTTTTCTGGAGCTCACCAATCTTTGTCTCGGTCATAACTTTCGGCACTTGTATATTGCTTGGTGGCGAGCTCACTGCCGGAGGTGT
TCTCTCTGCTTTAGCAACTTTTAGGATCCTTCAAGAGCCTCTGAGGAATTTCCCTGATCTCATCTCCATGATAGCTCAGACGAGGGTGT
CCTTGGACAGGTTGTCACACTTTTTGCGGCAAGAAGAGTTGCCGGATGATGCAACAATAAGTGTTCCACAGGGTAGCACAGACAAGGCA
ATCGATATCAAGGATGGCAGTTTCTCTTGGAACCCGTCTTGCTCAACCCCTACACTATCTCATATACAACTTAGTGTGGTGAGAGGCAT
GAGAGTAGCAGTCTGTGGTGTGATTGGCTCTGGCAAATCAAGTCTATTGTCCTCTATACTTGGGGAGATACCCAGACTGTCTGGCCAAG
TAAGTACATGCGTAAAAAAAGAGTTCATGCTAACTTGAGTGACCGTCTAATTACATTTTCCTTCTGCTCCTTATTCAGGTTAGGGTCAG
TGGTACAGCAGCATATGTTTCACAGACTGCCTGGATACAGTCTGGAAATATTGAGGAGAACGTTCTTTTCGGCACTCCAATGGACAGAC
CGCGCTATAAGAGAGTACTTGAGGCTTGCTCCCTGAAGAAGGATCTTCAGTTGCTCCAGTATGGTGATCAGACCATCATCGGTGACAGA
GGCATCAATTTGAGTGGAGGCCAGAAACAGAGAGTGCAGCTTGCGAGAGCACTGTACCAGGATGCTGATATTTATTTGCTTGATGACCC
CTTCAGTGCTGTTGATGCTCATACTGGAAGTGATCTATTTAAGGTCTGTATAGTTGTTGCCTTATTCTAGTTCTTTCTTGAAGGTGCTT
CTGTTTCGTAACATGTCAGCTTTTGTGTTGCAGGACTATATATTGGGGGCACTAGCTAGTAAAACAGTAATTTATGTAACTCATCAAGT
CGAGTTCCTACCAGCTGCTGACTTGATATTGGTAATAACATTTTTCTCACAATCTAAGCACTAGTACATTTAGTGGCTAATGTACATAG
ATATGCTGTTTCAAATTTCTAACAGCCTATTAAGTTGTTCAATTCGACATATTGTTTATCCATGTTTCTTAATGAGAATAAAACAGCAG
ATTCTGCATGAAAGTTTCGCTATCTTCTATGTACAATACTTCTGATAGGATAATGGGCCCTTGATCACTCGTTTGTGCTTTCTTTTTAT
CATGGCATAGGTTCTTAAGGATGGTCATATCACCCAAGCTGGAAAATATGATGATCTTCTCCAAGCTGGAACCGATTTCAATGCTCTGG
TTTCTGCTCACAATGAAGCTATCGAAACCATGGATTTCGGCGAAGATTCTGATGGAGATATTGCTCCTTCTGTTCCTAACAAAAGATTG
ACACCAAGTGTTAGCAATATCGATAACCTGAAAAATAAAGTATCTGAAAATGGTAAGTCATCTAATACACGTGGAATTAAGGACAAGAA
AAAGAGTGAAGAACGTAAGAAGAAGCGTACTGTTCAGGAAGAGGAGAGGGAGCGAGGAAGAGTTAGCTTAAATGTTTATTTGACATACA
TGGGGGAAGCCTACAAAGGTTCACTTATACCACTCATTGTCTTGGCACAAACCCTGTTCCAAGTTCTTCAGATTGCCAGTAACTGGTGG
ATGGCATGGGCAAACCCACAAACAGAAGGAGATGCACCTAAGACAAGTAGTGTGGTCCTTCTTGTTGTTTATATGTGCCTTGCTTTCGG
GAGTTCATTGTTTGTGTTTGTGAGAAGCCTTCTTGTAGCTACATTTGGTTTAGCAGCTGCTCAGAAACTATTTATAAAAATGCTAAGGT
GTGTGTTTCGAGCGCCAATGTCATTCTTCGATACTACACCATCTGGACGGATTCTGAATCGAGTAAGTTTAGATCATGCTCTTGCTCCT
AAATTGTGTTTTTCACTCTCGCGTGTGCCTTTTCTATTTTGCACATCCTAATCTTCTTCAATCATACTTCATATCAGGTTTCTGTAGAT

CAAAGTGTTGTGGACCTTGATATAGCATTCAGGCTAGGGGGATTTGCCTCAACAACAATTCAACTCCTTGGAATTGTTGCTGTCATGAG
TAAAGTCACATGGCAAGTTCTGTTTCTTATAGTCCCTATGGCTATGGCATGCATGTGGATGCAGGTAAATTGCTAAACTTTCAGGTTTC
TTTTAGTGTTGGCATTAACCTGGGATACTTAATTTTTTCAATGGAGATCGTAATGCAAGTAAAGTAGATAGCAAGGGTAGGTACACCTA
AATGAATCAATAAAAGAACTATGCGCGCATAAATTTGGCCACCTCTGTTAGTTAACTAGCCAATTAACCAGAAAACAGATGTATATGTT
CAGCATGAAAGTATTGAAACTTGTGAAGTAAGCTCATTCTTAAAGCATGAGCTTTCAAGATAGCTTGATGATTTATGTACCAAAGACTT
TTAAAGATCTTTATCATCACACAATAGTTAATGAATGTCCTTGCTACCATGAACTTGCAGAGATATTACATTGCTTCATCAAGGGAGCT
GACTAGGATCTTGAGTGTTCAGAAGTCTCCAGTGATCCATTTGTTTAGCGAGTCAATCGCTGGTGCTGCTACTATCAGAGGATTTGGTC
AGGAAAAAAGATTCATGAAAAGAAATCTTTACCTCCTTGATTGTTTTGCTCGGCCTTTGTTTTCCAGCCTTGCAGCTATTGAATGGCTC
TGCCTGCGAATGGAATTGCTCTCGACTTTTGTGTTTGCTTTCTGCATGGCAATACTCGTGAGCTTTCCTCCTGGAACAATTGAACCAAG
TATGTCTTATCTGCTATCTCACAGGCCGTTCTGTTACATTTTATGATCTTGCTTTACCCAATAGATCACTTGTTAGGTGCACTAGAAAA
CTTGCATATCTTTTTCAATAAAATATGGCAATGTGCTTCTCAATCTAAACAAGCTTTCCAATTGATAGGTATGGCTGGGCTTGCTGTCA
CATATGGACTCAACCTAAATGCTCGGATGTCAAGGTGGATATTGAGCTTCTGTAAATTAGAGAACAGAATCATCTCTGTTGAGCGTATT
TATCAGTACTGCAAGATTCCTAGTGAAGCACCACTGATTATTGAGAATTGTCGTCCCCCGGCCTCATGGCCTGAGAATGGAAACATTCA
ATTGATTGATCTCAAGGTAAACTTAGTCCGTAGCCATTTATTTGTGACTATCCATTTGCTCTTCGTTATACATAATCACCAGAAGTATA
TACTCTGTTTTTTCTTCTTCTAAGCTAAAAATACTTCAGTTTTATTTACGGCATCTTATTGTGCTTTTGCTAAATATCATAGGTCCGCT
ACAAGGACGACCTTCCCTTCGTTCTACATGGAGTCAGTTGTATTTTTCCTGGCGGGAAAAAGATTGGGATTGTAGGGAGAACTGGAAGT
GGTAAATCTACTCTCATTCAGGCTCTTTTCCGTCTAATTGAACCCACAGGAGGGAAAATTATCATTGACGACATCGATGTTTCTGCGAT
TGGCCTTCACGATCTGCGGTCACGGTTGAGCATCATCCCTCAGGACCCTACATTGTTCGAGGGTACTATCAGAATGAATCTTGACCCTC
TTGAAGAGCGTTCTGATCAAGAAATTTGGGAGGTACGCCATGGTCGCTTTTATGCTATCCTCATGTTAAATGTGCCATGATCTTTATGG
AAGAGAACTCTTTGTTTCAGGCACTAGAGAAGTGCCAGCTAGGAGAGGTCATTCGTTCGAAGGAAGAAAAACTGGACAGTCCAGGTTTG
TTCTAATTTTGAATATTCAAACACTTCTTCAAAGAGTGAGCATGACACTTGGTAAAGCATGACACCTCGTAAACAAAATTATATTTTCC
TGGATATTTGTGGTGCCATTGCAGTACTGGAGAATGGGGATAACTGGAGTGTGGGACAGCGCCAGCTTATTGCATTGGGTAGGGCGCTG
CTTAAGCAGGCAAGAATTTTGGTGCTTGATGAGGCGACGGCATCAGTTGACACAGCTACAGATAATCTTATCCAGAAGATTATTCGCAG
CGAATTCAGGGATTGCACTGTCTGTACAATTGCACACAGGATCCCAACGGTCATCGACAGTGACCTAGTCATGGTGCTTAGCGACGGTA
CATGCTCCTTCACACGATTAACCAAGTCATCTTTCCAGCCGTAGCTAGACCATTACATGTTTTGTTTCTTACTGATCGTATGTTGAATT
ATCTGAGTAACGCTCCTAACCAAACAGTGTTCTTTGTCACGCCCGAAAACTTGGGAGCAACAAACAGCGGTTACGGTGAATTCACCTGG
TCTTGTTTATTTTCCAGGTAAAATAGCGGAGTTCGACACACCCCAGAGGCTTGTGGAGGACAAGTCCTCCATGTTCATGCAGCTAGTAT
CCGAGTACTCCACCAGGGCGAGCTGTATATAGAGAGAGAGGCTTATTAGCTTAAATCCCCCACCGAGAATAGAGGCTGGGAGGTAAGGT
AGCCGGATCTGCCAGTCTACTCACACCATACAAGTAAGTAGCAACACCAGACAGGAGGATAGGAGGGGTATATGAACGGAAAGAAGCGC
CGGGCGCGTTCCCTGACCTGAATGGCCATGGATTCTTCTTCGAGGGAACGCGGCAGGCGATCGAACACCACGGGAGGAGTTGGCGGCAT
TAACCGAAGCTCTGATGCTTTTGTATGTATAACACTCTGTACTGCTTCTCCCCTTGCATGATGGGAAAACGAACACAAATAACGTGGGT
AATAAAGGGGGAAATTTTGCTTTC

>TdMRP3-B1 protein
MPRRALPLAEAAAAAAHAALLALALLLLLLRGARALASRCASCLKPPRRARNPALAGDGAPLAPSPPAAGGAWYRAALACCAYALLAQL
AALSYEVAAAAPPAEAEALLLPAVQALAWAALLALALRARAGRFPALVRVWWVLAFALSLAIAFDDSRRLMGADDHDADYAHMVANFAS
LPALGFLCLVGVMGSSGVDLEFSDDDTGVHEPLLLGGQRRGAEEEPGCLRVTPYGDAGILSLATLSWLSPLLSVGAKRPLELADIPLLA
HKDRAKFCYKAMSSHYERQRLECPDKEPSLAWAILKSFWREAAINGAFAAVNTVVSYVGPYLISYFVDYLSGKIAFPHEGYILASVFFV
SKLIETLTARQWYLGVDVMGIHVKSGLTAMVYRKGLRLSNASKQSHTSGEIVNYMAVDVQRVGDYAWYFHDIWMLPLQIILALAILYKN
VGIATVSTLIATALSIAASVPVAKLQEHYQDKLMAAKDERMRKTAECLKSMRILKLQAWEDRYRIMLEEMRNVECRWLKWALYSQAAVT
FVFWSSPIFVSVITFGTCILLGGELTAGGVLSALATFRILQEPLRNFPDLISMIAQTRVSLDRLSHFLRQEELPDDATISVPQGSTDKA
IDIKDGSFSWNPSCSTPTLSHIQLSVVRGMRVAVCGVIGSGKSSLLSSILGEIPRLSGQVRVSGTAAYVSQTAWIQSGNIEENVLFGTP
MDRPRYKRVLEACSLKKDLQLLQYGDQTIIGDRGINLSGGQKQRVQLARALYQDADIYLLDDPFSAVDAHTGSDLFKDYILGALASKTV
IYVTHQVEFLPAADLILVLKDGHITQAGKYDDLLQAGTDFNALVSAHNEAIETMDFGEDSDGDIAPSVPNKRLTPSVSNIDNLKNKVSE
NGKSSNTRGIKDKKKSEERKKKRTVQEEERERGRVSLNVYLTYMGEAYKGSLIPLIVLAQTLFQVLQIASNWWMAWANPQTEGDAPKTS
SVVLLVVYMCLAFGSSLFVFVRSLLVATFGLAAAQKLFIKMLRCVFRAPMSFFDTTPSGRILNRVSVDQSVVDLDIAFRLGGFASTTIQ
LLGIVAVMSKVTWQVLFLIVPMAMACMWMQRYYIASSRELTRILSVQKSPVIHLFSESIAGAATIRGFGQEKRFMKRNLYLLDCFARPL
FSSLAAIEWLCLRMELLSTFVFAFCMAILVSFPPGTIEPSMAGLAVTYGLNLNARMSRWILSFCKLENRIISVERIYQYCKIPSEAPLI
IENCRPPASWPENGNIQLIDLKVRYKDDLPFVLHGVSCIFPGGKKIGIVGRTGSGKSTLIQALFRLIEPTGGKIIIDDIDVSAIGLHDL
RSRLSIIPQDPTLFEGTIRMNLDPLEERSDQEIWEALEKCQLGEVIRSKEEKLDSPVLENGDNWSVGQRQLIALGRALLKQARILVLDE
ATASVDTATDNLIQKIIRSEFRDCTVCTIAHRIPTVIDSDLVMVLSDGKIAEFDTPQRLVEDKSSMFMQLVSEYSTRASCI

Supplementary Figure S2. Genomic sequences of TdMRP3 homeoalleles and their deduced amino acid sequences.Exons are highlighted in light grey.

MSA

The multiple sequence alignment result as produced by T-coffee.

```
T-COFFEE, Version_11.00 (Version_11.00)Cedric Notredame SCORE=985
*
 BAD AVG GOOD
*

TdMRP3-A   :  97
TdMRP3-B   :  97
TaMRP3-A   :  97
TaMRP3-B   :  97
TaMRP3-D   :  97
HvMRP4     :  99
ZmMRP4     :  97
OsMRP5     :  97
AtMRP5     :  95
cons       :  98

TdMRP3-A    II RL-RA-----------HTPLPLTEAAAAAAHAALLALALLLLLLRGARALASRCASCLKP PRRAR ---
TdMRP3-B    II RR-----------------ALPLAEAAAAAAHAALLALALLLLLLRGARALASRCASCLKP-PRRARNPA-
TaMRP3-A    II RL-RA-----------HTPLPLTEAAAAAAHAALLALALLLLLLRGARALASRCASCLKP-PR RARN---
TaMRP3-B    II RR ---------------- ALPLAEAAAAAAHAALLALALLLLLLRGARALASRCASCLKP-PR RARNPA-
TaMRP3-D    II LHFQVQPPVLQLQDYCYYYQQ QQEAAAAAAHAALLALALLLLLLRGARALASRCASCLKP-PRRARN---
HvMRP4
ZmMRP4      MI PS-F ------------ PSLPL EAVAATAHAALLALAALLLLLRAARALASRCASCLKA-PRRRGGPAV
OsMRP5      MI H--F ------------ PNLPL EAAAAAAHAALLALALLLLLLRSARALASRCASCLKTAPRRAA---A
AtMRP5   MD    FI-EISLIF ------ REHLPLLELCSVIINLLLFLVFL ------ FAVSARQILVCV R-GRDRL----

cons

TdMRP3-A    LVHDGPPLA -------PPPAAGGAWFRAALACCAYVLLAQLAALTYEVAAAPPP-VEAEALLLPAVQALAW
TdMRP3-B    LAGDGAPLAP ------ SPPAAGGAWYRAALACCAYALLAQLAALSYEVAAAAPP-AEAEALLLPAVQALAW
TaMRP3-A    LVHDGPPLAS ------ PPPAAGGAWFRAALACCAYVLLAQLAALTYEVAAAPPP-VEAEALLLPAVQALAW
TaMRP3-B    LAGDGAPLAP ------ SPPAAGGAWYRAALACCAYALLAQLAALSYEVAAAAPP-AEAEPLLLPAVQALAW
TaMRP3-D    LVHDGPPLAS ------ PPPAAGGAWFRAALACCAYALLAQLAALTYEVAAAAPP-VEAEALLLPAVQALAW
HvMRP4                                                                          --
ZmMRP4      VVGDGAGGAL ------- AAATAGAWHRAVLASCAYALLSQVAVLSYEVAVAGSR-VSARALLLPAVQAVSW
OsMRP5      --VDGGLAA --------- ASSVGAWYRAALACCGYALLAQVAALSYEVAVAGSH-VAVEALLLPAVQALAW
AtMRP5      -SKDDTVSASNLSLEREVNHVSVGFGFNLSLLCCLYVLGVQVLVLVYDGVKVRREVSDWFVLCFPASQSLAW

cons
```

TDM0

```
TdMRP3-A    AALLALALRARAGGRGRFPALVRVWWVLAFALSVAIAFDDSRRLMGAD-DRDADYAHMVANFASLPALGFLC
TdMRP3-B    AALLALALRARA---GRFPALVRVWWVLAFALSLAIAFDDSRRLMGAD-DHDADYAHMVANFASLPALGFLC
TaMRP3-A    AALLALALRARAGGRGRFPALVRVWWVLAFALSVAIAFDDSRRLMGAD-DRDADYAHMVANFASLPALGFLC
TaMRP3-B    AALLALALRARA---GRFPALVRVWWVLAFALSLAIAFDDSRRLMGAD-DHDADYAHMVANFASLPALGFLC
TaMRP3-D   AALLALALRARA---GRFPALVRVWWVLAFALSVAIAFDDSRRLMGAD-DRDADYAHMVANFASLPALGFLC
HvMRP4      -------------------------------------------MGGA-DHDVDYAHMVANFASLPALGFLC
ZmMRP4      AALLALALQARAVGWARFPALVRLWWVVSFALCVVIAYDDSRRLIGQG-ARAVDYAHMVANFASVPALGFLC
OsMRP5      AALLALAMQARAVGWGRFPVLVRVWWVVSFVLCVGIAYDDTRHLMGDDDDDEVDYAHMVANFASAPALGFLC
AtMRP5     FVLSFLVLHLKYKSSEKLPFLVRIWWFLAFSICLCTMYVDGRRLAIE--GWSRCSSHVVANLAVTPALGFLC

cons                                                            :*:***:*   *******

TdMRP3-A    LVGVMGSSGVELEFSDDDTGVHEPLLLGGQRRGAEEEPGCLRVTPYGDAGILSLATLSWLSPLLSVGAKRPL
TdMRP3-B    LVGVMGSSGVDLEFSDDDTGVHEPLLLGGQRRGAEEEPGCLRVTPYGDAGILSLATLSWLSPLLSVGAKRPL
TaMRP3-A    LVGVMGSSGVELEFSDDDTGVHEPLLLGGQRRGAEEEPGCLRVTPYGDAGILSLATLSWLSPLLSVGAKRPL
TaMRP3-B    LVGVMGSSGVDLEFSDDDTGVHEPLLLGGQRRGAEEEPGCLRVTPYGDAGILSLATLSWLSPLLSVGAKRPL
TaMRP3-D    LVGVMGSSGVDLEFSDDDTGVHEPLLLGGQRRGAEEEPGCLRVTPYGDAGILSLATLSWLSPLLSVGAKRPL
HvMRP4      LVGVMGSSGVELEFSDDDTGVHEPLLLGGQRRDAEEEPGCLRVTPYGDAGILSLATLSWLSPLLSVGAKRPL
ZmMRP4      LVGVMGSTGLELEFTEDGNGLHEPLLLGRQRREAEEELGCLRVTPYADAGILSLATLSWLSPLLSVGAQRPL
OsMRP5      LVGVMGSTGVELEFTDDDSSVHEPLLLGGQRRDADEEPGCLRVTPYGDAGIVSLATLSWLSPLLSVGAQRPL
AtMRP5     FLAWRGVSGIQVTR--SSSDLQEPLLVEE      EAACLKVTPYSTAGLVSLITLSWLDPLLSAGSKRPL

cons        ::.  * :*:::    ....::****:    * .**:****. **::** *****.****.*::***
```

```
TdMRP3-A    ELADIPLLAHKDRAKFCYKAMSSHYERQRLECPDKEPSLAWAILKSFWREAAINGAFAAVNTVVSYVGPYLI
TdMRP3-B    ELADIPLLAHKDRAKFCYKAMSSHYERQRLECPDKEPSLAWAILKSFWREAAINGAFAAVNTVVSYVGPYLI
TaMRP3-A    ELADIPLLAHKDRAKFCYKAMSSHYERQRLECPDKEPSLAWAILKSFWREAAINGAFAAVNTVVSYVGPYLI
TaMRP3-B    ELADIPLLAHKDRAKFCYKAMSSHYERQRLECPDKEPSLAWAILKSFWREAAINGAFAAVNTVVSYVGPYLI
TaMRP3-D    ELADIPLLAHKDRAKFCYKAMSSHYERQRLECPDKEPSLAWAILKSFWREAAMNGAFAAVNTVVSYVGPYLI
HvMRP4      ELADIPLLAHKDRAKFCYKAMSSHYERQRIECPDKEPSLAWAILKSFWREAAINGCFAAVNTVVSYVGPYLI
ZmMRP4      ELADIPLLAHKDRAKSCYKAMSAHYERQRLEYPGREPSLTWAILKSFWREAAVNGTFAAVNTIVSYVGPYLI
OsMRP5      ELADIPLMAHKDRAKSCYKAMSSHYERQRMERPGSEPSLAWAILKSFWREAAINGAFAAVNTIVSYVGPYLI
AtMRP5  ELKDIPLLAPRDRAKSSYKVLKSNWKRCKSENPSKPPSLARAIMKSFWKEAACNAVFAGLNTLVSYVGPYLI

cons        ** ****:* :**** .**.:.::::* : * *.  ***: **:****:*** *. **.:**:*********


TdMRP3-A    SYFVDYLSGKIAFPHEGYILASVFFVSKLIETLTARQWYLGVDVMGIHVKSGLTAMVYRKGLRLSNASKQSH
TdMRP3-B    SYFVDYLSGKIAFPHEGYILASVFFVSKLIETLTARQWYLGVDVMGIHVKSGLTAMVYRKGLRLSNASKQSH
TaMRP3-A    SYFVDYLSGKIAFPHEGYILASVFFVSKLIETLTARQWYLGVDVMGIHVKSGLTAMVYRKGLRLSNASKQSH
TaMRP3-B    SYFVDYLSGKIAFPHEGYILASVFFVSKLIETLTARQWYLGVDVMGIHVKSGLTAMVYRKGLRLSNASKQSH
TaMRP3-D    SYFVDYLSGKIAFPHEGYILASVFFVSKLIETLTARQWYLGVDVMGIHVKSGLTAMVYRKGLRLSNASKQSH
HvMRP4      SYFVDYLSGKIAFPHEGYILASVFFVSKLIETLTARQWYLGVDVMGIHVKSGLTAMVYRKGLRLSNASKQSH
ZmMRP4      SYFVDYLSGNIAFPHEGYILASIFFVAKLLETLTARQWYLGVDIMGIHVKSGLTAMVYRKGLRLSNASRQSH
OsMRP5      SYFVDYLSGKIEFPHEGYILASVFFVAKLLETLTARQWYLGVDVMGIHVKSGLTAMVYRKGLRLSNSSRQSH
AtMRP5  SYFVDYLGGKEIFPHEGYVLAGIFFTSKLIETVTTRQWYMGVDILGMHVRSALTAMVYRKGLKLSSIAKQNH

cons        *******.*:  ******:**.:**.:**:**:*:****:***::*:**:*.**********:**. ::*.*


TdMRP3-A    TSGEIVNYMAVDVQRVGDYAWYFHDIWMLPLQIILALAILYKNVGIATVSTLIATALSIAASVPVAKLQEHY
TdMRP3-B    TSGEIVNYMAVDVQRVGDYAWYFHDIWMLPLQIILALAILYKNVGIATVSTLIATALSIAASVPVAKLQEHY
TaMRP3-A    TSGEIVNYMAVDVQRVGDYAWYFHDIWMLPLQIILALAILYKNVGIATVSTLIATALSIAASVPVAKLQEHY
TaMRP3-B    TSGEIVNYMAVDVQRVGDYAWYFHDIWMLPLQIILALAILYKNVGIATVSTLIATALSIAASVPVAKLQEHY
TaMRP3-D    TSGEIVNYMAVDVQRVGDYAWYFHDIWMLPLQIILALAILYKNVGIATVSTLIATALSIAASVPVAKLQEHY
HvMRP4      TSGEIVNYMAVDVQRVGDYAWYFHDIWMLPLQIILALAILYKNVGIATVSTLIATALSIAASVPVAKLQEHY
ZmMRP4      TSGEIVNYMAVDVQRVGDYAWYFHDIWMLPLQIILALAILYKNVGIAMVSTLVATVLSIAASVPVAKLQEHY
OsMRP5      TSGEIVNYMAVDVQRVGDYAWYFHDIWMLPLQIILALAILYKNVGIAMVSTLVATVLSIAASVPVAKLQEHY
AtMRP5  TSGEIVNYMAVDVQRIGDYSWYLHDIWMLPMQIVLALAILYKSVGIAAVATLVATIISILVTIPLAKVQEDY

cons        ***************:***:**:*******:**:*********.**** *:**:** :** .::*:**:**.*
                                IPR011527 ABCC1_TM_dom

TdMRP3-A    QDKLMAAKDERMRKTAECLKSMRILKLQAWEDRYRIMLEEMRNVECRWLKWALYSQAAVTFVFWSSPIFVSV
TdMRP3-B    QDKLMAAKDERMRKTAECLKSMRILKLQAWEDRYRIMLEEMRNVECRWLKWALYSQAAVTFVFWSSPIFVSV
TaMRP3-A    QDKLMAAKDERMRKTAECLKSMRILKLQAWEDRYRIMLEEMRNVECRWLKWALYSQAAVTFVFWSSPIFVSV
TaMRP3-B    QDKLMAAKDERMRKTAECLKSMRILKLQAWEDRYRIMLEEMRNVECRWLKWALYSQAAVTFVFWSSPIFVSV
TaMRP3-D    QDKLMAAKDERMRKTAECLKSMRILKLQAWEDRYRIMLEEMRNVECRWLKWALYSQAAVTFVFWSSPIFVSV
HvMRP4      QDKLMAAKDERMRKTAECLKSMRILKLQAWEDRYRIMLEEMRNVECRWLKWALYSQAAVTFVFWSSPIFVSV
ZmMRP4      QDKLMASKDERMRKTSECLKNMRILKLQAWEDRYRLQLEEMRNVECRWLRWALYSQAAVTFVFWSSPIFVAV
OsMRP5      QDKLMASKDERMRKTSECLKNMRILKLQAWEDRYRLKLEEMRNVECKWLRWALYSQAAVTFVFWSSPIFVAV
AtMRP5  QDKLMTAKDERMRKTSECLRNMRVLKLQAWEDRYRVRLEEMREEEYGWLRKALYSQAFVTFIFWSSPIFVAA

cons        *****::********:***:.**:***********: *****: *  **: ****** ***:********:.


TdMRP3-A    ITFGTCILLGGELTAGGVLSALATFRILQEPLRNFPDLISMIAQTRVSLDRLSHFLRQEELPDDATISVPQG
TdMRP3-B    ITFGTCILLGGELTAGGVLSALATFRILQEPLRNFPDLISMIAQTRVSLDRLSHFLRQEELPDDATISVPQG
TaMRP3-A    ITFGTCILLGGELTAGGVLSALATFRILQEPLRNFPDLISMIAQTRVSLDRLSHFLRQEELPDDATISVPQG
TaMRP3-B    ITFGTCILLGGELTAGGVLSALATFRILQEPLRNFPDLISMIAQTRVSLDRLSHFLRQEELPDDATISVPQG
TaMRP3-D    ITFGTCILLGGELTAGGVLSALATFRILQEPLRNFPDLISMIAQTRVSLDRLSHFLRQEELPDDATISVPQG
HvMRP4      ITFGTCILLGGELTAGGVLSALATFRILQEPLRNFPDLISMIAQTRVSLDRLSHFLRQEELPDDATISVPQG
ZmMRP4      ITFGTCILLGGQLTAGGVLSALATFRILQEPLRNFPDLISMMAQTRVSLDRLSHFLQQEELPDDATINVPQS
OsMRP5      ITFGTCILLGGELTAGGVLSALATFRILQEPLRNFPDLISMIAQTRVSLDRLSHFLQQEELPDDATITVPHG
AtMRP5  VTFATSIFLGTQLTAGGVLSALATFRILQEPLRNFPDLVSMMAQTKVSLDRISGFLQEEELQEDATVVIPRG

cons        :**.*.*:** :*************************:**:***:*****:* **::*** :***: :*:.
                                                          NBD1 (Walker A)

TdMRP3-A    STDKAIDIRDGSFSWNPSCSNPTLSDIQLSVVRGMRVAVCGVIGSGKSSLLSSILGEIPKLSGQVRISGTAA
TdMRP3-B    STDKAIDIKDGSFSWNPSCSTPTLSHIQLSVVRGMRVAVCGVIGSGKSSLLSSILGEIPRLSGQVRVSGTAA
TaMRP3-A    STDKAIDIRDGSFSWNPSCSNPTLSDIQLSVVRGMRVAVCGVIGSGKSSLLSSILGEIPKLSGQVRISGTAA
TaMRP3-B  STDKAIDIKDGSFSWNPSCSTPTLSHIQLSVVRGMRVAVCGVIGSGKSSLLSSILGEIPRLSGQVRVSGTAA
```

```
TaMRP3-D   STDKAIDIKDGSFSWNPSCSTPTLSHIQLSVVRGMRVAVCGVIGSGKSSLLSSILGEIPRLSGQVRVSGTAA
HvMRP4     STDKAIDIRDGNFSWNPSCSTPTLYGIQLSVVRGMRVAVCGVIGSGKSSLLSSILGEIPRLSGQVRVSGTAA
ZmMRP4     STDKAVDIKDGAFSWNPYTLTPTLSDIHLSVVRGMRVAVCGVIGSGKSSLLSSILGEIPKLCGHVRISGTAA
OsMRP5     STDKAININDATFSWNPSSPTPTLSGINLSVVRGMRVAVCGVIGSGKSSLLSSILGEIPKLCGQVRISGSAA
AtMRP5    LSNIAIEIKDGVFCWDPFSSRPTLSGIQMKVEKGMRVAVCGTVGSGKSSFISCILGEIPKISGEVRICGTTG

cons       :: *::*.*. *.*:*     ***  *::.* :********.:******::*.******::.*.**:.*::.
                                 IPR003593 AAA+ ATPase

TdMRP3-A   YVSQTAWIQSGNIEENVLFGTPMDRPRYKRVLEACSLKKDLQLLQYGDQTIIGDRGINLSGGQKQRVQLARA
TdMRP3-B   YVSQTAWIQSGNIEENVLFGTPMDRPRYKRVLEACSLKKDLQLLQYGDQTIIGDRGINLSGGQKQRVQLARA
TaMRP3-A   YVSQTAWIQSGNIEENVLFGTPMDRPRYKRVLEACSLKKDLQLLQYGDQTIIGDRGINLSGGQKQRVQLARA
TaMRP3-B   YVSQTAWIQSGNIEENVLFGTPMDRPRYKRVLEACSLKKDLQLLQYGDQTIIGDRGINLSGGQKQRVQLARA
TaMRP3-D   YVSQTAWIQSGNIEENVLFGTPMDRPRYKRVLEACSLKKDLQLLQYGDQTIIGDRGINLSGGQKQRVQLARA
HvMRP4     YVSQTAWIQSGNIEENVLFGTPMDRPRYKRVLEACSLKKDLQLLQYGDQTIIGDRGINLSGGQKQRVQLARA
ZmMRP4     YVPQTAWIQSGNIEENILFGSQMDRQRYKRVIAACCLKKDLELLQYGDQTVIGDRGINLSGGQKQRVQLARA
OsMRP5     YVPQTAWIQSGNIEENILFGSPMDKQRYKRVIEACSLKKDLQLLQYGDQTIIGDRGINLSGGQKQRVQLARA
AtMRP5    YVSQSAWIQSGNIEENILFGSPMEKTKYKNVIQACSLKKDIELFSHGDQTIIGERGINLSGGQKQRVQLARA

cons       **.*:***********:***: *:: :**.*: **.****::*:.:****:**:*****************
                Walker B

TdMRP3-A   LYQDADIYLLDDPFSAVDAHTGSDLFKDYILGALASKTVIYVTHQVEFLPAADLILVLKDGHITQAGKYDDL
TdMRP3-B   LYQDADIYLLDDPFSAVDAHTGSDLFKDYILGALASKTVIYVTHQVEFLPAADLILVLKDGHITQAGKYDDL
TaMRP3-A   LYQDADIYLLDDPFSAVDAHTGSDLFKDYILGALASKTVIYVTHQVEFLPAADLILVLKDGHITQAGKYDDL
TaMRP3-B   LYQDADIYLLDDPFSAVDAHTGSDLFKDYILGALASKTVIYVTHQVEFLPAADLILVLKDGHITQAGKYDDL
TaMRP3-D   LYQDADIYLLDDPFSAVDAHTGSDLFKDYILGALASKTVIYVTHQVEFLPAADLILVLKDGHITQAGKYDDL
HvMRP4     LYQDADIYLLDDPFSAVDAHTGSDLFKDYILGALASKTVIYVTHQVEFLPAADLILVLKDGRITQAGKYDDL
ZmMRP4     LYQDADIYLLDDPFSAVDAHTGSELFKEYILTALATKTVIYVTHQVEFLPAADLILVLKDGHITQAGKYDDL
OsMRP5     LYQDADIYLLDDPFSAVDAHTGSELFREYILTALASKTVIYVTHQIEFLPAADLILVLKDGHITQAGKYDDL
AtMRP5    LYQDADIYLLDDPFSALDAHTGSDLFRDYILSALAEKTVVFVTHQVEFLPAADLILVLKEGRIIQSGKYDDL

cons       ***************:******:**::*** *** ***::****:*************:*:* *:******

                                                                 Disorder region
TdMRP3-A   LQAGTDFNALVSAHNEAIETMDFGEDSDGDIAPSVPNKRLTP ------ SVSNIDNLKNKVSENGKSSNTRG
TdMRP3-B   LQAGTDFNALVSAHNEAIETMDFGEDSDGDIAPSVPNKRLTP ------ SVSNIDNLKNKVSENGKSSNTRG
TaMRP3-A   LQAGTDFNALVSAHNEAIETMDFGEDSDGDIAPSVPNKRLTP ------ SVSNIDNLKNKVSENGKSSNTRG
TaMRP3-B   LQAGTDFNALVSAHNEAIETMDFGEDSDGDIAPSVPNKRLTP ------ SVSNIDNLKNKVSENGKSSNTRG
TaMRP3-D   LQAGTDFNALVSAHNEAIETMDFGEDSDGDIAPSVPNKRLTP ------ SVSNIDNLKNKVSENGKSSNTRG
HvMRP4     LQAGTDFNALVSAHNEAIETMDFGEDSDGDIAPSVPNKRLIP ------ SVSNIDNLKNKVSENGKSSNTRG
ZmMRP4     LQAGTDFNALVSAHKEAIETMDIFEDSDSDTVSSIPNKRLTP ------ SISNIDNLKNKMCENGQPSNTRG
OsMRP5     LQAGTDFNALVCAHKEAIETMEFSEDSDEDTVSSVPIKRLTP ------ SVSNIDNLKNKVSNNEKPSSTRG
AtMRP5     LQAGTDFKALVSAHHEAIEAMDIPSPSSEDSDENPIRDSLVLHNPKSDVFENDIETLAKEVQEGGSASDLKA

cons       *******:***.**:****:*::  . *.  *     .    . *        .:*:.* ::: :.  ..*. :.

             Basic and acid residues
TdMRP3-A   IKDK-KKSEERKKKRTVQEEERERGRVSLNVYLTYMGEAYKGSLIPLIVLAQTLFQVLQIASNWWMAWANPQ
TdMRP3-B   IKDK-KKSEERKKKRTVQEEERERGRVSLNVYLTYMGEAYKGSLIPLIVLAQTLFQVLQIASNWWMAWANPQ
TaMRP3-A   IKDK-KKSEERKKKRTVQEEERERGRVSLNVYLTYMGEAYKGSLIPLIVLAQTLFQVLQIASNWWMAWANPQ
TaMRP3-B   IKDK-KKSEERKKKRTVQEEERERGRVSLNVYLTYMGEAYKGSLIPLIVLAQTLFQVLQIASNWWMAWANPQ
TaMRP3-D   IKDK-KKSEERKKKRTVQEEERERGRVSLNVYLTYMGEAYKGSLIPLIVLAQTLFQVLQIASNWWMAWANPQ
HvMRP4     IKDK-KKSEERKKKRTVQEEERERGRVSLNVYLTYMGEAYKGSLIPLIVLAQTLFQVLQIASNWWMAWANPQ
ZmMRP4     IKEK-KKKEERKKKRTVQEEERERGKVSSKVYLSYMGEAYKGTLIPLIILAQTMFQVLQIASNWWMAWANPQ
OsMRP5     IKEKKKKPEERKKKRSVQEEERERGRVSLQVYLSYMGEAYKGTLIPLIILAQTMFQVLQIASNWWMAWANPQ
AtMRP5   IKEKKKKAKRSRKKQLVQEEERVKGKVSMKVYLSYMGAAYKGALIPLIILAQAAFQFLQIASNWWMAWANPQ

cons       **:* ** :. :**: ****** :*:** :***:*** ****:*****:***: **.***************
                          IPR044726 ABCC_6TM_D2

TdMRP3-A   TEGDAPKTSSVVLLVVYMCLAFGSSLFVFVRSLLVATFGLAAAQKLFIKMLRCVFRAPMSFFDTTPSGRILN
TdMRP3-B   TEGDAPKTSSVVLLVVYMCLAFGSSLFVFVRSLLVATFGLAAAQKLFIKMLRCVFRAPMSFFDTTPSGRILN
TaMRP3-A   TEGDAPKTSSVVLLVVYMCLAFGSSLFVFVRSLLVATFGLAAAQKLFIKMLRCVFRAPMSFFDTTPSGRILN
TaMRP3-B   TEGDAPKTSSVVLLVVYMCLAFGSSLFVFVRSLLVATFGLAAAQKLFIKMLRCVFRAPMSFFDTTPSGRILN
TaMRP3-D   TEGDAPKTSSVVLLVVYMCLAFGSSLFVFVRSLLVATFGLAAAQKLFIKMLRCVFRAPMSFFDTTPSGRILN
HvMRP4     TEGDTPKTSSVVLLVVYMCLAFGSSLFVFVRSLLVATFGLAAAQKLFIKMLRCVFRAPMSFFDTTPSGRILN
ZmMRP4     TEGDAPKTDSVVLLVVYMSLAFGSSLFVFMRSLLVATFGLAAAQKLFIKMLRCVFRAPMSFFDTTPSGRILN
OsMRP5   TEGDAPKTDSVVLLVVYMSLAFGSSLFVFVRSLLVATFGLATAQKLFVKMLRCVFRAPMSFFDTTPSGRILN
```

AtMRP5	TEGDESKVDPTLLLIVYTALAFGSSVFIFVRAALVATFGLAAAQKLFLNMLRSVFRAPMSFFDSTPAGRILN
cons	**** .*....:**:** .******:*:*:*: ********:*****::***.**********:**:*****
TdMRP3-A	**RVSVDQSVVDLDIAFRLGGFASTTIQLLGIVAVMSKVTWQVLFLIVPMAMACMWMQRYYIASSRELTRILSV**
TdMRP3-B	**RVSVDQSVVDLDIAFRLGGFASTTIQLLGIVAVMSKVTWQVLFLIVPMAMACMWMQRYYIASSRELTRILSV**
TaMRP3-A	RVSVDQSVVDLDIAFRLGGFASTTIQLLGIVAVMSKVTWQVLFLIVPMAMACMWMQRYYIASSRELTRILSV
TaMRP3-B	RVSVDQSVVDLDIAFRLGGFASTTIQLLGIVAVMSKVTWQVLFLIVPMAMACMWMQRYYIASSRELTRILSV
TaMRP3-D	RVSVDQSVVDLDIAFRLGGFASTTIQLLGIVAVMSKVTWQVLFLIVPMAMACMWMQRYYIASSRELTRILSV
HvMRP4	RVSVDQSVVDLDIAFRLGGFASTTIQLLGIVAVMSKVTWQVLFLIVPMAMACMWMQRYYIASSRELTRILSV
ZmMRP4	RVSVDQSVVDLDIAFRLGGFASTTIQLLGIVAVMSKVTWQVLILIVPMAVACMWMQRYYIASSRELTRILSV
OsMRP5	RVSVDQSVVDLDIAFRLGGFASTTIQLLGIVAVMSKVTWQVLILIVPMAVACMWMQRYYIASSRELTRILSV
AtMRP5	RVSIDQSVVDLDIPFRLGGFASTTIQLCGIVAVMTNVTWQVFLLVVPVAVACFWMQKYYMASSRELVRIVSI
cons	***:*********.************* ******::*****::*:**:*:**:***:**:******.**:*:
TdMRP3-A	**QKSPVIHLFSESIAGAATIRGFGQEKRFMKRNLYLLDCFARPLFSSLAAIEWLCLRMELLSTFVFAFCMAIL**
TdMRP3-B	**QKSPVIHLFSESIAGAATIRGFGQEKRFMKRNLYLLDCFARPLFSSLAAIEWLCLRMELLSTFVFAFCMAIL**
TaMRP3-A	QKSPVIHLFSESIAGAATIRGFGQEKRFMKRNLYLLDCFARPLFSSLAAIEWLCLRMELLSTFVFAFCMAIL
TaMRP3-B	QKSPVIHLFSESIAGAATIRGFDQEKRFMKRNLYLLDCFARPLFSSLAAIEWLCLRMELLSTFVFAFCMAIL
TaMRP3-D	QKSPVIHLFSESIAGAATIRGFGQEKRFMKRNLYLLDCFARPLFSSLAAIEWLCLRMELLSTFVFAFCMAIL
HvMRP4	QKSPVIHLFSESIAGAATIRGFGQEKRFMKRNLYLLDCFARPLFSSLAAIEWLCLRMELLSTFVFAFCMAIL
ZmMRP4	QKSPVIHLFSESIAGAATIRGFGQEKRFMKRNLYLLDCFARPLFSSLAAIEWLCLRMELLSTFVFAFCMAIL
OsMRP5	QKSPVIHLFSESIAGAATIRGFGQEKRFMKRNLYLLDCFARPLFSSLAAIEWLCLRMELLSTFVFAFCMAIL
AtMRP5	QKSPIIHLFGESIAGAATIRGFGQEKRFIKRNLYLLDCFVRPFFCSIAAIEWLCLRMELLSTLVFAFCMVLL
cons	****:****.************.*****:**********.**:*.*:***************:******.:*
TdMRP3-A	**VSFPPGTIEPSMAGLAVTYGLNLNARMSRWILSFCKLENRIISVERIYQYC**KIPSEAPLIIENCRPPSSWPE
TdMRP3-B	**VSFPPGTIEPSMAGLAVTYGLNLNARMSRWILSFCKLENRIISVERIYQYC**KIPSEAPLIIENCRPPASWPE
TaMRP3-A	VSFPPGTIEPSMAGLAVTYGLNLNARMSRWILSFCKLENRIISVERIYQYCKIPSEAPLIIENCRPPSSWPE
TaMRP3-B	VSFPPGTIEPSMAGLAVTYGLNLNARMSRWILSFCKLENRIISVERIYQYCKIPSEAPLIIENCRPPASWPE
TaMRP3-D	VSFPPGTIEPSMAGLAVTYGLNLNARMSRWILSFCKLENRIISVERIYQYCKIPSEAPLIIENCRPPSSWPE
HvMRP4	VSFPPGTIEPSMAGLAVTYGLNLNARMSRWILSFCKLENRIISVERIYQYCKIPSEAPLIIENCRPPSSWPE
ZmMRP4	VSFPPGTIEPSMAGLAVTYGLNLNARMSRWILSFCKLENRIISVERIYQYCRLPSEAPLIIENCRPPSSWPQ
OsMRP5	VSFPPGTIEPSMAGLAVTYGLNLNARMSRWILSFCKLENRIISVERIYQYCKLPSEAPLIIENSRPSSSWPE
AtMRP5	VSFPHGTIDPSMAGLAVTYGLNLNGRLSRWILSFCKLENKIISIERIYQYSQIVGEAPAIIEDFRPPSSWPA
cons	**** ***:***************.*:************:***:******.:: .*** ***: **.:***
	NBD2
TdMRP3-A	NGNIELI**DLKVRYKDDLPFVLHGVSCIFPGGKKIGIV*GRTGSGKS*TLIQALFRLIEPAGGKIIIDNIDASAI**
TdMRP3-B	NGNIQLI**DLKVRYKDDLPFVLHGVSCIFPGGKKIGIV*GRTGSGKS*TLIQALFRLIEPTGGKIIIDDIDVSAI**
TaMRP3-A	NGNIELIDLKVRYKDDLPFVLHGVSCIFPGGKKIGIVGRTGSGKSTLIQALFRLIEPAGGKIIIDNIDASAI
TaMRP3-B	NGNIQLIDLKVRYKDDLPFVLHGVSCIFPGGKKIGIVGRTGSGKSTLIQALFRLIEPTGGKIIIDDIDVSAI
TaMRP3-D	NGNIELIDLKVRYKDDLPFVLHGVSCIFPGGKKIGIVGRTGSGKSTLIQALFRLIEPSGGKIIIDNIDVSAI
HvMRP4	NGNIELIDLKVRYKDDLPFVLHGVSCIFPGGKKIGIVGRTGSGKSTLIQALFRLIEPAGGKIIIDDIDVSAI
ZmMRP4	NGNIELIDLKVRYKDDLPLVLHGVSCMFPGGKKIGIVGRTGSGKSTLIQALFRLIEPTGGKIIIDNIDISAI
OsMRP5	NGNIELVDLKVRYKDDLPLVLHGISCIFPGGKKIGIVGRTGSGKSTLIQALFRLIEPTGGKVIIDDVDISRI
AtMRP5	TGTIELVDVKVRYAENLPTVLHGVSCVFPGGKKIGIVGRTGSGKSTLIQALFRLIEPTAGKITIDNIDISQI
cons	.*.*:*:*:**** ::** ****:**:*****************************:.**: **::* * *
	**IPR003593_AAA+_ATPase**
TdMRP3-A	**GLHDLRSRLSIIPQDPTLFEGTIRMNLDPLEERSDQEIWEALEKCQLGEVIRSKEEKLDSPVLENGDNWSVG**
TdMRP3-B	**GLHDLRSRLSIIPQDPTLFEGTIRMNLDPLEERSDQEIWEALEKCQLGEVIRSKEEKLDSPVLENGDNWSVG**
TaMRP3-A	GLHDLRSRLSIIPQDPTLFEGTIRMNLDPLEERSDQEIWEALEKCQLGEVIRSKEEKLDSPVLENGDNWSVG
TaMRP3-B	GLHDLRSRLSIIPQDPTLFEGTIRMNLDPLEERSDQEIWEALEKCQLGEVIRSKEEKLDSPVLENGDNWSVG
TaMRP3-D	GLHDLRSRLSIIPQDPTLFEGTIRMNLDPLEERSDQEIWEALEKCQLGEVIRSKEEKLDSPVLENGDNWSVG
HvMRP4	GLHDLRSRLSIIPQDPTLFEGTIRMNLDPLEERSDQEIWEALEKCQLGEVIRSKEEKLDSPVLENGDNWSVG
ZmMRP4	GLHDLRSRLSIIPQDPTLFEGTIRMNLDPLEECTDQEIWEALEKCQLGEVIRSKEEKLDSPVLENGDNWSVG
OsMRP5	GLHDLRSRLSIIPQDPTLFEGTIRMNLDPLEECTDQEIWEALEKCQLGEVIRSKDEKLDSPVLENGDNWSVG
AtMRP5	GLHDLRSRLGIIPQDPTLFEGTIRANLDPLEEHSDDKIWEALDKSQLGDVVRGKDLKLDSPVLENGDNWSVG
cons	*********.************** ******* :*::*****:*.***:*:*.*: ****************

```
TdMRP3-A   QRQLIALGRALLKQARILVLDEATASVDTATDNLIQKIIRSEFRDCTVCTIAHRIPTVIDSDLVMVLSDGKI
TdMRP3-B   QRQLIALGRALLKQARILVLDEATASVDTATDNLIQKIIRSEFRDCTVCTIAHRIPTVIDSDLVMVLSDGKI
TaMRP3-A   QRQLIALGRALLKQARILVLDEATASVDTATDNLIQKIIRSEFRDCTVCTIAHRIPTVIDSDLVMVLSDGKI
TaMRP3-B  QRQLIALGRALLKQARILVLDEATASVDTATDNLIQKIIRSEFRDCTVCTIAHRIPTVIDSDLVMVLSDGKI


TaMRP3-D   QRQLIALGRALLKQARILVLDEATASVDTATDNLIQKIIRSEFRDCTVCTIAHRIPTVIDSDLVMVLSDGKI
HvMRP4     QRQLIALGRALLKQARILVLDEATASVDTATDNLIQKIIRSEFRDCTVCTIAHRIPTVIDSDLVMVLSDGKI
ZmMRP4     QRQLIALGRALLKQAKILVLDEATASVDTATDNLIQKIIRSEFKDCTVCTIAHRIPTVIDSDLVLVLSDGKI
OsMRP5     QRQLIALGRALLKQAKILVLDEATASVDTATDNLIQKIIRSEFKDCTVCTIAHRIPTVIDSDLVLVLSDGKI
AtMRP5   QRQLVSLGRALLKQAKILVLDEATASVDTATDNLIQKIIRTEFEDCTVCTIAHRIPTVIDSDLVLVLSDGRV

cons       ****::*********:************************:**.*******************:*****::


TdMRP3-A   AEFDTPQRLLEDKSSMFMQLVSEYSTRASCI---
TdMRP3-B   AEFDTPQRLVEDKSSMFMQLVSEYSTRASCI---
TaMRP3-A   AEFDTPQRLLEDKSSMFMQLVSEYSTRASCI---
TaMRP3-B   AEFDTPQRLVEDKSSMFMQLVSEYSTRASCI---
TaMRP3-D   AEFDTPQRLVEDKSSMFMQLVSEYSTRASCI---
HvMRP4     AEFDTPQRLLEDKSSMFMQLVSEYSTRASCI---
ZmMRP4     AEFDTPQRLLEDKSSMFIQLVSEYSTRSSCI---
OsMRP5     AEFDTPQRLLEDKSSMFMQLVSEYSTRSSCI---
AtMRP5   AEFDTPARLLEDKSSMFLKLVTEYSSRSTGIPEL

cons       ****** **:*******::**:***:*:: *
```

Supplementary Figure S3. Multiple sequence alignment (MSA) of the ABCC multidrug resistance associated protein (ABCC-MRP) in different plant species. Td: *Triticum turgidum* ssp. *durum* MRP3-A1, MRP3-B1, Ta: *Triticum aestivum* MRP3-A (TraesCS5A02G512500), MRP3-B (TraesCS4B02G343800), MRP3-D (TraesCS4D02G339000); Hv: *Hordeum vulgare* MRP4 (XP 044983147.1); Zm: *Zea mays* MRP4 (EF586878); Os: *Oryza sativa Japonica* group MRP5 (XP 015630971.1); At: *Arabidopsis thaliana* MRP5 (AT1G04120.1). The MSA alignment is coloured according to the T-Coffee TCS scheme.Dark pink bits are very reliable, while blue and green bits are unreliable based on the T-Coffee library.

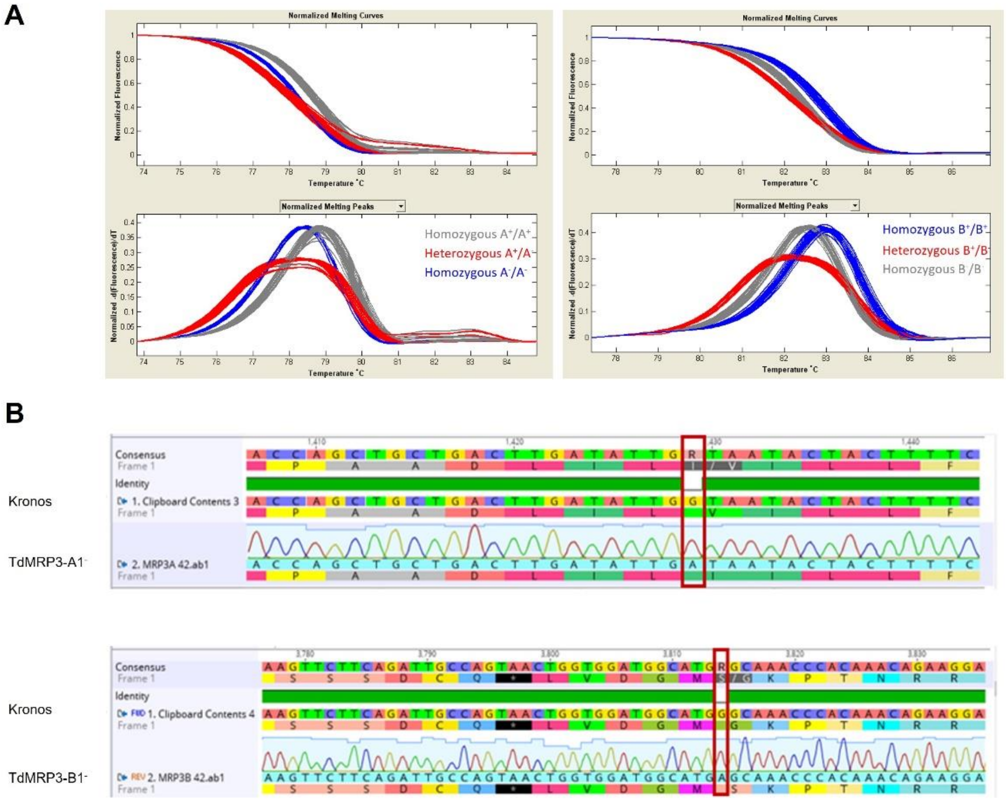

Supplementary Figure S4. Selection of the mutant genotypes by MAS and Sanger sequencing. (A) HRM genotyping of F_2 and F_3 progenies from the cross TdMRP3-A1$^-$ x TdMRP3-B1$^-$. The different melting curve profiles show the heterozygous plants and homozygous mutant or wild type lines. On the left HRM analysis of the *TdMRP3-A1* homeoallele; on the right HRM analysis of the *TdMRP3-B1* homeoallele. (B) Example of the sequencing of a double null homozygous mutant for the *TdMRP3* genes selected by HRM genotyping in the F_2 progeny. On the top sequences related to the *TdMRP3-A1* homeoallele. On the bottom sequences of the *TdMRP3-B1*.

A

B

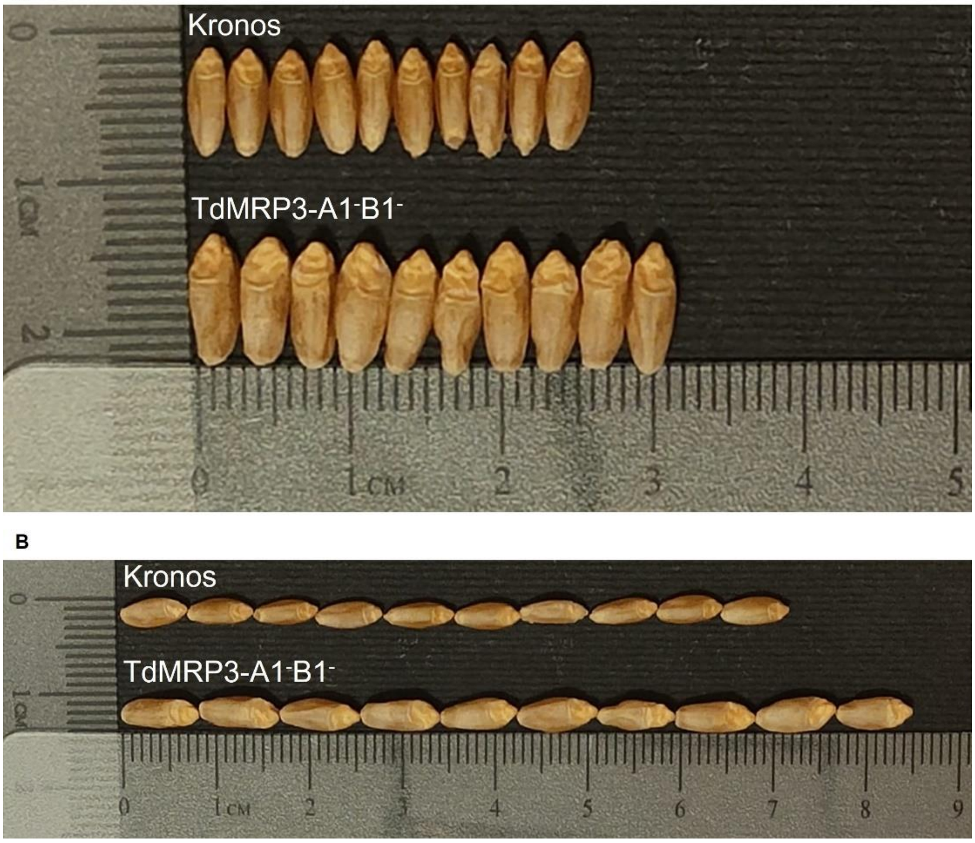

Supplementary Figure S5. Grain size comparison between the control (cv. Kronos) and the complete null TdMRP3 mutant lines.

Supplementary Table S1. List of primers used for HRM genotyping.

ID primer	Sequence	T Annealing	Amplicon Size	Use	Genome
**MRP3A_F**	TTGCGAGAGCATTGTACCAAG	58°C	460bp	1st round PCR	A
**MRP3A_R**	CATGAACAGTCCAATGAGAAG				
**MRP3Aex3F1**	AACCCATCAAGTCGAGTTCC	60°C	89bp	2nd round PCR	
**MRP3Aex3R**	GAACAACTCAATAGGCTAATAG				
**MRP3B_F**	ATGTACAATACTTCTGATAGG	58°C	780bp	1st round PCR	B
**MRP3B_R**	GAAAAGGCACACGCAAGAGTG				
**4443_F**	GTTCCAAGTTCTTCAGATTG	60°C	92bp	2nd round PCR	
**4443_R2**	GGACCACACTACTTGTCTTA				

Supplementary Table S2. ICP-MS operating conditions.

ICP-MS parameters	**Setting**

RF power	1550 W
RF Matching	1.80 V
Carrier gas flow rate (Argon)	0.90 L min^{-1}
Dilution Mode	ON
Dilution Gas (Argon)	0.30 L min^{-1}
Sampling depth	8.0 mm
S/C temp	2°C
Collision Gas	He
Collision gas flow rate	4.0 L min^{-1} (*10 L min^{-1})
Isotope monitored	^{23}Na, ^{24}Mg, ^{39}K, ^{44}Ca, ^{55}Mn, ^{59}Co, ^{56}Fe, ^{60}Ni, ^{63}Cu, ^{66}Zn, ^{78}Se*, ^{95}Mo
Internal standards	^{6}Li, ^{45}Sc, ^{72}Ge, ^{89}Y, ^{115}In, ^{159}Tb
Integration Time/Mass	0.3 sec
